# Dual‐Working‐Pattern Nanosheet‐Based Hydrogel Sensors for Constructing Human‐Machine and Physiological‐Electric Interfaces

**DOI:** 10.1002/advs.202414301

**Published:** 2025-06-29

**Authors:** Shitao Shi, Yuanyuan Wang, Zewei Ye, Hongxia Xie, Chencong Liu, Jiaqi Liao, Dawei Zhao, Qingfeng Sun, Julia L. Shamshina, Xiaoping Shen

**Affiliations:** ^1^ College of Chemistry and Materials Engineering Zhejiang A&F University Hangzhou 311300 China; ^2^ Key Laboratory on Resources Chemicals and Materials of Ministry of Education Shenyang University of Chemical Technology Shenyang Liaoning Province 110142 China; ^3^ Fiber and Biopolymer Research Institute Department of Plant and Soil Science Texas Tech University Lubbock TX 79409 USA; ^4^ Department of Chemistry and Biochemistry Texas Tech University Lubbock TX 79409 USA

**Keywords:** cellulose nanosheets, conductive paths, dual‐working‐pattern, epidermal electronics, microcapacitor arrays

## Abstract

While hydrogels are ideal building blocks for fabricating next‐generation epidermal electronics to acquire high‐fidelity electrical signals induced by motion and physiological activities, an unresolved issue remains: the differentiation and selection of sensing modes in hydrogel sensors. The novel design leverages numerous conductive nanosheets, randomly arranged in a series‐parallel configuration, embedded within a highly compliant dielectric hydrogel. For the nanosheets, poly(3,4‐ethylenedioxythiophene) (PEDOT) is deposited on the surface of sulfonated cellulose nanosheets (SCNS) to function as microelectrodes (PEDOT@SCNS). The resulting nanosheet‐based hydrogel (NSH) demonstrates remarkable stretchability (1356%), excellent adaptability (storage modulus of 102 Pa), and self‐adhesiveness (21.7 kPa on pigskin). The nanosheet microelectrodes enable the formation of both microcapacitor arrays and conductive paths within the ultrasoft hydrogel, facilitating the construction of high‐fidelity capacitive sensors and bioelectrodes for the real‐time monitoring and classification of human activities and physiological states, respectively. This NSH, which significantly reduces skin‐interfacial impedance, has demonstrated strong potential as candidate sensors for advanced applications in EMG, facial nerve monitoring, ECG, and brain activity monitoring, achieving reduced RMS noise (9.7 µV) and minimal motion artifacts.

## Graphical abstract

Hydrogels hold great potential as candidates for next‐generation epidermal electronics; however, an unresolved issue remains: the differentiation and selection of sensing modes in hydrogel sensors. This study integrates series‐parallel conductive nanosheet configurations into an ultra‐soft hydrogel, resulting in enhanced signal intensity and conformally stable physiological‐electronic interfaces when used as motion and electrophysiological sensors in dual‐working‐patterned wearable electronics.

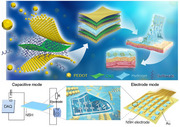

## 1 Introduction

Epidermal electronics are electronic devices that have elastic moduli, thicknesses, bending stiffnesses, and areal mass densities to integrate well with the epidermis.^[^ ^1^ ^]^ Proper physical properties enable these devices to conform to the relief on their surface and interface softly and noninvasively, even with neonatal skin.^[^ ^2^ ^]^ An efficient on‐skin‐wearable mechanosensor is capable of accurately recording biopotential signals, such as electrocardiography (ECG or EKG), electromyography (EMG), and electroencephalography (EEG), for monitoring physiological parameters in real‐time, as well as diagnosing and treating heart‐, brain‐, and muscle‐related conditions.^[^ ^3^ ^]^ It also facilitates multifunctional control of artificial intelligence machinery^[^ ^4^ ^]^ and even enhances the functionality of patient prostheses.^[^ ^5^ ^]^ Epidermal electronics utilizing flexible hydrogels are promising next‐generation wearable platforms that facilitate real‐time monitoring of physiological and physical signals for healthcare and human‐machine interface (HMI) applications.^[^ ^6^ ^]^ Depending on the material, hydrogel electrodes may offer biocompatibility, along with enhanced conductivity, as well as adjustable modulus and adhesiveness and skin‐conformability, which would minimize biomechanical mismatch at tissue‐electrode interfaces and optimize the signal recording quality by facilitating compliant, seamless, and firm integration with the skin.^[^ ^7^ ^]^

The mechanically compliant hydrogel can form seamless integration between the bioelectronics and human skins, tissues, etc., providing soft mechanical coupling, low interfacial impedance, efficient signal exchange, and minimal motion artifacts, while also reducing tissue damage and inflammation response.^[^ ^3^, ^8^ ^]^ To enable hydrogels to self‐adapt and conform to the skin's texture, including wrinkles, creases, and pits, with an average roughness up to ≈190 µm,^[^ ^9^ ^]^ it is crucial to develop hydrogels with low moduli (below 1–100 kPa, which is the modulus range of soft biological tissues).^[^ ^10^ ^]^ The softness and stretchability of hydrogels require the incorporation of dynamic covalent or non‐covalent cross‐linking including ionic,^[^ ^11^ ^]^ hydrogen,^[^ ^12^ ^]^ borate ester,^[^ ^13^ ^]^ Schiff base,^[^ ^14^ ^]^ host‐guest,^[^ ^15^ ^]^ Diels‐Alder,^[^ ^16^ ^]^ and disulfide bonds^[^ ^17^ ^]^ into networks with relatively low cross‐linking density.^[^ ^18^ ^]^ However, conductive phases often have intrinsic limitations that hinder self‐compliance, such as high Young's modulus or restricted deformability. Most stretchable hydrogel electrodes are created by incorporating electronically conducting nanofillers such as silver flakes,^[^ ^19^ ^]^ graphene nanosheets,^[^ ^20^ ^]^ 2D early transition metal carbides and nitrides or MXenes,^[^ ^21^ ^]^ metal ions,^[^ ^22^ ^]^ and conductive polymers^[^ ^23^ ^]^ into a soft polymeric matrix. These designs, therefore, face a trade‐off between conductivity and shape adaptability.

Herein, we propose an innovative strategy for developing a hydrogel enriched with conductive nanosheets that exhibit high compliance with skin, thereby improving sensitivity and establishing stable bioelectronic interfaces. The conductive composite nanosheets are synthesized via in situ oxidative polymerization of 3,4‐ethylenedioxythiophene (EDOT) monomers on sulfonated cellulose nanosheets with a degree of sulfonation (DS) of 0.16. The resulting organic PEDOT@SCNS nanosheets, which function as microelectrodes, are uniformly dispersed within a dual‐crosslinked carboxymethyl cellulose/polyacrylamide (CMC/PAAm) soft hydrogel matrix. This arrangement creates a distributed, series‐parallel interconnected network of internal microcapacitors and conductive pathways. This work provides a systematic exploration of the mechanisms underlying the sensing capabilities and self‐adaptability of the hydrogel, with the goal of achieving efficient mechanosensing performance. The proposed nanosheet‐based hydrogel (NSH) HMIs can function as strain sensors (either capacitive or piezoresistive) and also serve as physiological electrodes on interdigitated electrode current collector arrays for versatile healthcare monitoring and motion‐controlled artificial intelligence applications. Both configurations demonstrate low skin impedance at physiologically relevant frequencies, high sensitivity, a low signal‐to‐noise ratio (SNR), and a small root‐mean‐square (RMS) noise value of 9.7 µV.

## 2 Results

### 2.1 Design Principle of Nanosheet‐Based Hydrogels

**Figure 1:**
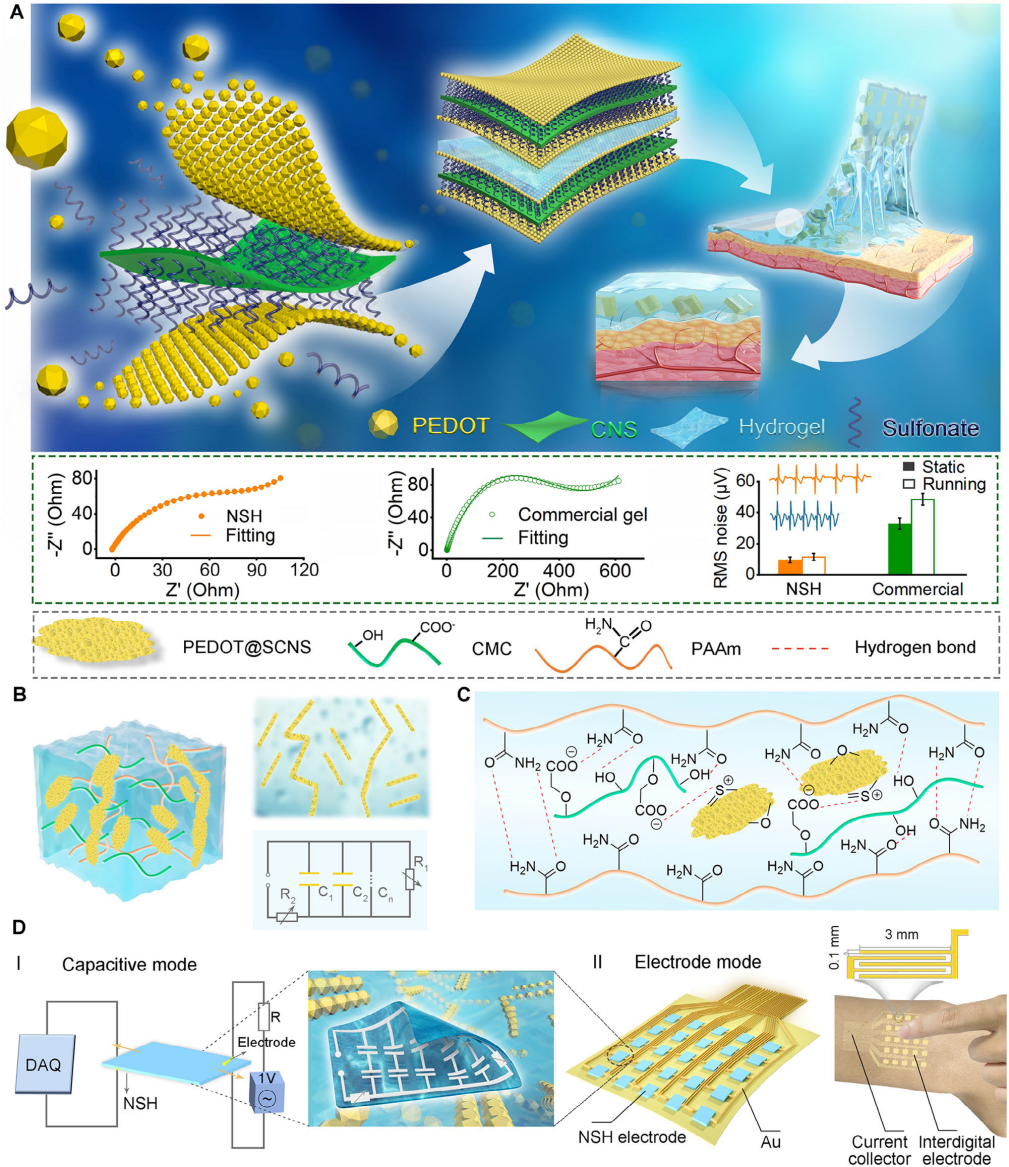
Schematic illustration of the fabrication of NSH for multimodal, highly sensitive and self‐adaptive bioelectronic interfaces. A) The concept of series‐parallel connected microcapacitors composed of 2D conductive cellulose nanosheets confined within a dielectric hydrogel for low‐impedance and high‐fidelity epidermal electronics. B) Schematic diagram of series‐parallel circuits of microcapacitors and conductive pathways. C) Uniform dispersion of the nanosheets and abundant interactions within the hydrogel. D) The dual working pattens of NSH: (I) capacitive mode under high‐frequency AC voltage, (II) resistive or electrode mode under DC voltage.

The concept of embedding conductive bio‐nanosheet configurations within a soft, stretchable, and highly compliant hydrogel matrix, as illustrated in **Figure** **1**A, aims to establish series‐parallel networks of microcapacitors and conductive pathways (Figure 1B). This approach thereby facilitates the development of multimodal, flexible mechanosensors that enable stable, high‐resolution bioelectronic interfaces. The 2D ultrathin conductive cellulose nanosheets function as the microscale electrodes, while the hydrogel acts as the dielectric layer. The preparation of the sandwich‐like composite conductive nanosheets involves three key steps: 1. dissolution, regeneration, and shear treatment of cellulose to obtain cellulose nanosheets (CNS); 2. surface sulfonation of CNSs to obtain sulfonated cellulose nanosheets (SCNS); and 3. in situ oxidative polymerization of 3,4‐ethylenedioxythiophene (EDOT) using ferric chloride (FeCl_3_) as the oxidant with the formation of PEDOT on both top and bottom surfaces of SCNS (PEDOT@SCNS). The negatively charged sulfonate groups on SCNS serve as charge compensators, balancing the positively charged π‐conjugated thiophene rings of Fe^3+^‐oxidized PEDOT.^[^ ^24^ ^]^ This dual functionality allows the sulfonate groups to act as both templates and doping sites for the polymerization of EDOT monomers.^[^ ^25^ ^]^ Additionally, the sulfonate groups on SCNS function as the counterions that help maintain the stability of the PEDOT dispersions (Figure S1, Supporting Information) and enhance the dispersibility and stability of the nanosheets within the PAAm/CMC hydrogel through the electrostatic interactions (Figure S2, Supporting Information).

The microstructure of the interpenetrated hydrogel can be significantly altered by regulating the concentration of macromolecules and thus the cross‐linking density. Thus, scanning electron microscopy (SEM) images of the freeze‐dried hydrogels reveal a homogeneous interconnected porous architecture (Figure S3, Supporting Information), where the pore diameters are directly influenced by the macromolecular content of PAAm. Specifically, when the acrylamide monomer is added at a concentration below 2 m, the hydrogel demonstrates a sparse and porous microstructure. Conversely, employing a 3 m acrylamide monomer concentration results in a hydrogel exhibiting a more densely packed matrix. This level of precise control over the porous structure is particularly useful for the development of hydrogels with tailored properties such as stretchability, flexibility, softness, compliance, and even adhesiveness.

Given the abundance of functional groups in components such as PEDOT@SCNS, CMC, and PAAm within the hydrogel, it is likely that a tight interaction between these components develop through physical hydrogen bonding and electrostatic associations (Figure 1C),^[^ ^13^, ^26^ ^]^ contributing to the uniform dispersion and mechanical stability of the composite network. As demonstrated in Figure S4 (Supporting Information), the resulting NSH exhibits excellent deformability, self‐compliance, and self‐adhesiveness, enabling it to adhere securely to any area of the skin surface, even during joint movements.

Ultimately, the abundant PEDOT@SCNS embedded within the NSH create numerous series‐parallel microcapacitive and conductive circuits throughout the hydrogel (Figure 1D). In an alternating electric field, the impedance (Z) of the microcapacitors is defined as Z = 1 / 2πfC, where f is the frequency of the AC source and C is the capacitance. As frequency of the AC source increases, the impedance of microcapacitors decreases, facilitating easier measurement of the capacitive signal. Under DC voltage, the microcapacitor array reaches a steady state, exerting no influence on the current flow. As a result, the NSH functions as a passive component. In summary, novel NSH is able to function under both AC and DC conditions and supports two versatile operational modes, acting either as a conductor or a piezoresistive sensor. This makes it highly promising for HMI arrays and epidermal electronics on multichannel interdigitated electrode current collectors, enabling real‐time, precise acquisition of biomotion and physiological signals.

### 2.2 Self‐Compliance of Hydrogels

**Figure 2:**
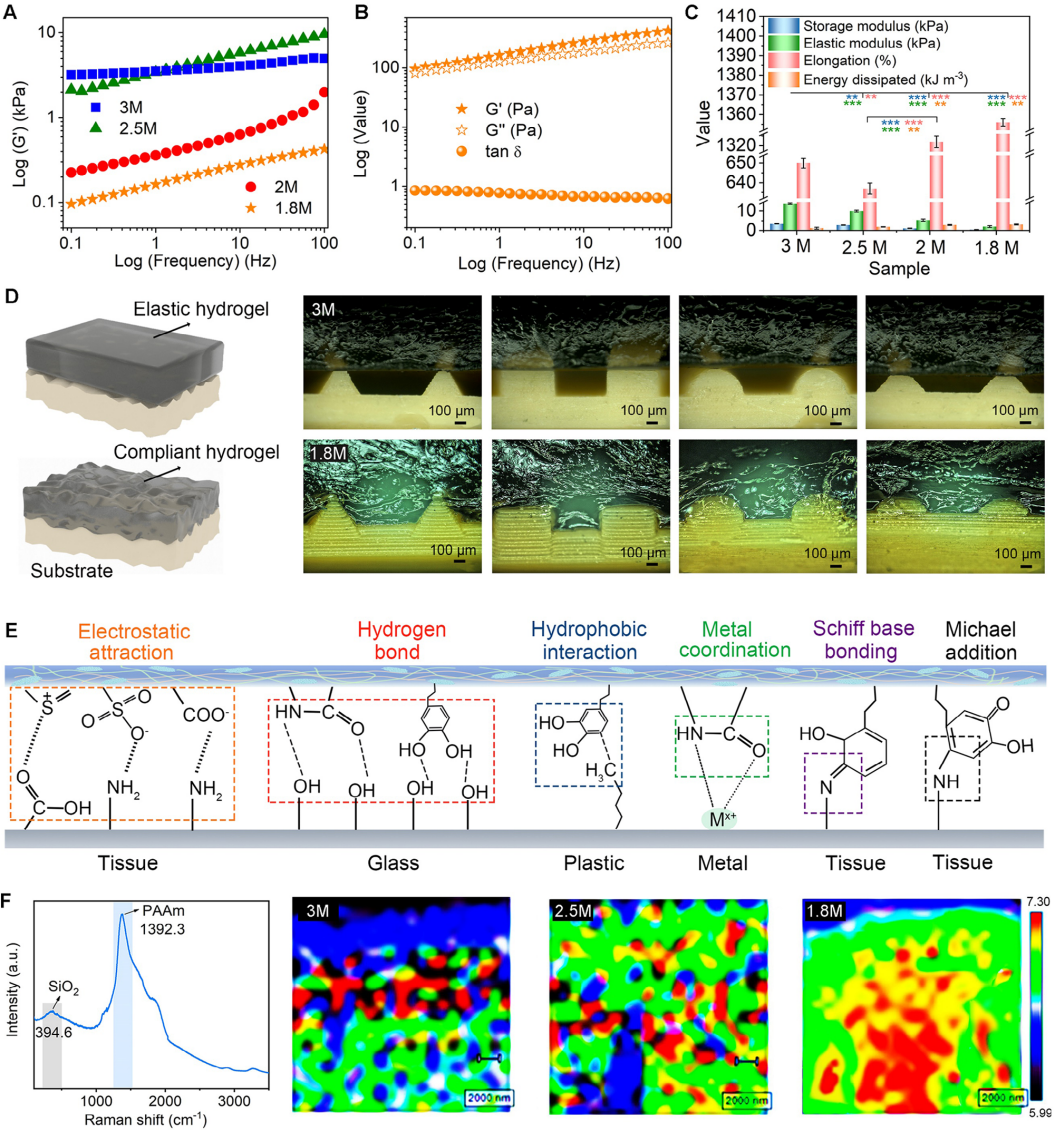
Self‐compliance and self‐adhesiveness of NSH for robust interface. A) Storage moduli of NSH with different matrix concentration. B) Frequency‐independent gel point state (loss factor tan δ ≈ 1) of the 1.8 m NSH. C) Comparison of mechanical property parameters. D) Schematic of the rheology‐based self‐compliance. Stereomicroscopic images showing the 3 m hydrogel (top) and 1.8 m hydrogel (bottom) placed on various patterned resin molds for 10 s. E) Possible interfacial interactions between the hydrogel and the substrate. Please noted that the structure of pDA has, to a certain degree, undergone simplification.^[^ ^29^ ^]^ F) 2D Raman images depicting the intensity ratio of the 1392 cm^−1^ peak to the 395 cm^−1^ peak for i) 3 m hydrogel, ii) 2.5 m hydrogel, and iii) 1.8 m hydrogel.

The storage modulus (G') of NSH increases with the AAm concentration, as demonstrated by the dynamic frequency rheological sweep test performed for hydrogels made with AAM concentration from 1.8 to 3 m (**Figure** **2**A). This indicates the formation of an increasingly dense crosslinked network within the hydrogel. More importantly, at a concentration of 1.8 M NSH reaches a critical state known as the gel point characterized by a loss factor (tan δ) of ≈1 (where tan δ = G′′/G′). This condition occurs over a wide frequency range (Figure 2B; Figure S5, Supporting Information), and indicates that the storage modulus (G′, which reflects the material's elasticity) is equal to the loss modulus (G′′, which reflects the material's viscosity). The low G' at this gel point indicates that the hydrogel possesses a high degree of viscoelasticity and softness in the 1.8 m hydrogel.^[^ ^27^ ^]^ Importantly, in this state, the hydrogel can dissipate strain energy during dynamic movements, allowing for better compliance with the skin and surrounding tissues. At the same time, it can recover durably, preventing free flow and maintaining its structure when not under stress.^[^ ^28^ ^]^ Therefore, the 1.8 m hydrogel is considered an ideal electrode interface, as it reduces contact damage between soft living tissues and attached bionic devices while ensuring interface stability.

The mechanical property parameters listed in Figure 2C, Figures S6 and S7 (Supporting Information) demonstrate that all NSH samples possess high stretchability and low hysteresis implying that the energy loss during stretching and releasing cycles is minimal. The NSH samples dissipate energy within the range of 10^1^ kJ m^−3^ during ten cycles of stretching at a 500% extension. The negligible hysteresis observed during stretching, even at large amplitudes and fixed rates, is likely due to the transmission of tension through entanglements between the chains. This allows multiple chains to respond to the applied force simultaneously. The interconnected network effectively distributes stress, preventing localized weaknesses that could lead to embrittlement. Additionally, this structure facilitates self‐resilience after a certain waiting time, enabling the material to recover its original properties following deformation. Specifically, for the 1.8 m NSH, the parameters indicate ultralow Young's moduli of ≈ 1 kPa, G' below 0.5 kPa, and tan δ ≤ 1. These values meet the criteria for remarkable “super‐compliance” without allowing free flow.

The extra‐soft and ultralow‐modulus hydrogel is capable of conforming to different substrates, regardless of their roughness or wrinkling, thereby maximizing the contact area (Figure 2D) and enhancing adhesion. The adaptability of the hydrogel is influenced by its entanglement crosslinking density, as shown in the cross‐sectional images from stereomicroscopy. For the 3 m hydrogel, its low compliance prevents spontaneous deformation along the patterned surface of molds. In contrast, the low‐concentration hydrogels demonstrate an ability to fill the grooves of varying morphologies on the patterned molds' surface without any gaps. This is because the low‐concentration NSH contains fewer intertwined chains, allowing it to be more flexible and adaptable. As a result, the lower‐concentration hydrogels achieve mechanical interlocking with substrates, increasing the effective contact area at the interface.

These soft, viscoelastic, stretchable, and conductive hydrogels can rapidly adhere to a wide variety of solid substrates including rubber, wood, polytetrafluoroethylene (PTFE), polypropylene (PP), and glass (Figure S8A,B, Supporting Information). The quantitative lap shear tests reveal varied adhesive strength on wood, ranging from 9.8 ± 2.2 kPa for the 3 m hydrogel to 25.2 ± 3.5 kPa for the 1.8 m sample (Figure S8C, Supporting Information). The adhesion strengths of the 1.8 m hydrogel to pigskin, plastic, aluminum (Al), and glass are 16.1 ± 4 kPa, 15.7 ± 2.5 kPa, 15.0 ± 1 kPa, and 12.5 ± 1.5 kPa, respectively, consistently exceeding 10 kPa for all substrates.

The adhesive performance is both repeatable and stable over time, as demonstrated by the 30 cycles of peeling and re‐adhering without losing effectiveness (Figure S8D, Supporting Information). This durability is attributed to the simultaneous formation of numerous physical and/or chemical anchoring sites on the substrates which depend on the surface chemistry of the materials they are bonding to (Figure 2E).^[^ ^29^ ^]^ The possible adhesive mechanisms include several interactions: electrostatic attraction, hydrogen bonding, metal coordination, hydrophobic interaction, and specific chemical reactions such as Schiff base formation and Michael addition. These interactions enable the hydrogels to adhere to a vatiety of surfaces encountered in daily life including biological tissue, glass, plastic, and metals.The presence of functional groups in the hydrogel, such as –SO_3_H^−^, –COO^−^, –C(O)NH–, and catechol groups in NSH, which exhibit high chemical reactivity or electron transfer capability, enhances these adhesive properties.^[^ ^18^, ^30^ ^]^

We infer that the rheological characteristics of NSH exert a greater influence on interfacial adhesion than chemical adhesion effects. To verify and illustrate the hydrogels' adhesion behavior on a glass substrate, 2D Raman mapping is employed (Figure 2F). The technique highlights their conformability and self‐adhesiveness. In the analysis, the intensity ratio of the 1392.3 cm⁻¹ peak (associated with PAAm in hydrogel) to the 394.6 cm⁻¹ peak (linked to SiO₂ in glass) is calculated and compared among hydrogels with varying levels of entanglement after quick in situ adhesion and subsequent detachment. It is observed that the adhesion of the 3 M hydrogel displays extensive blue areas (indicating a low‐intensity ratio) alongside localized red areas (indicating a high‐intensity ratio). In contrast, the interface formed by the 1.8 m hydrogel shows a notable shift toward red. This shift indicates that the intensity ratio significantly increases across the interface when using the low‐concentration hydrogel, with a more widespread distribution. The enhanced performance of the 1.8 m hydrogel can be attributed to its ability to readily deform and conform to the substrate.

To further enhance NSH adhesion while maintaining its mechanical and electrical properties, specific biopolymers such as gelatin, chitosan, lignin, as well as synthetic polymers such as *N*‐[tris(hydroxymethyl)methyl] acrylamide (THAM), polyethyleneimine (PEI), and polydopamine (pDA) are incorporated into the hydrogel network (Table S1, Supporting Information, Supporting Information). Among these compositions, those containing gelatin and pDA demonstrate the highest adhesive strengths, achieving values of 29.2 and 27.1 kPa on wood, and 21.7 and 20.4 kPa on pigskin, respectively (Figure S8E, Supporting Information). These adhesion strengths are eitherhigher than or comparable to many adhesive ionogels and hydrogels reported in the literature.^[^ ^31^ ^]^ Moreover, when the hydrogel is peeled off from human skin, it leaves an ultrathin residue layer, which can be easily removed with water and ethanol without causing any harm to the skin (Figure S9, Supporting Information).

### 2.3 Microcapacitive Sensing Mechanism

**Figure 3:**
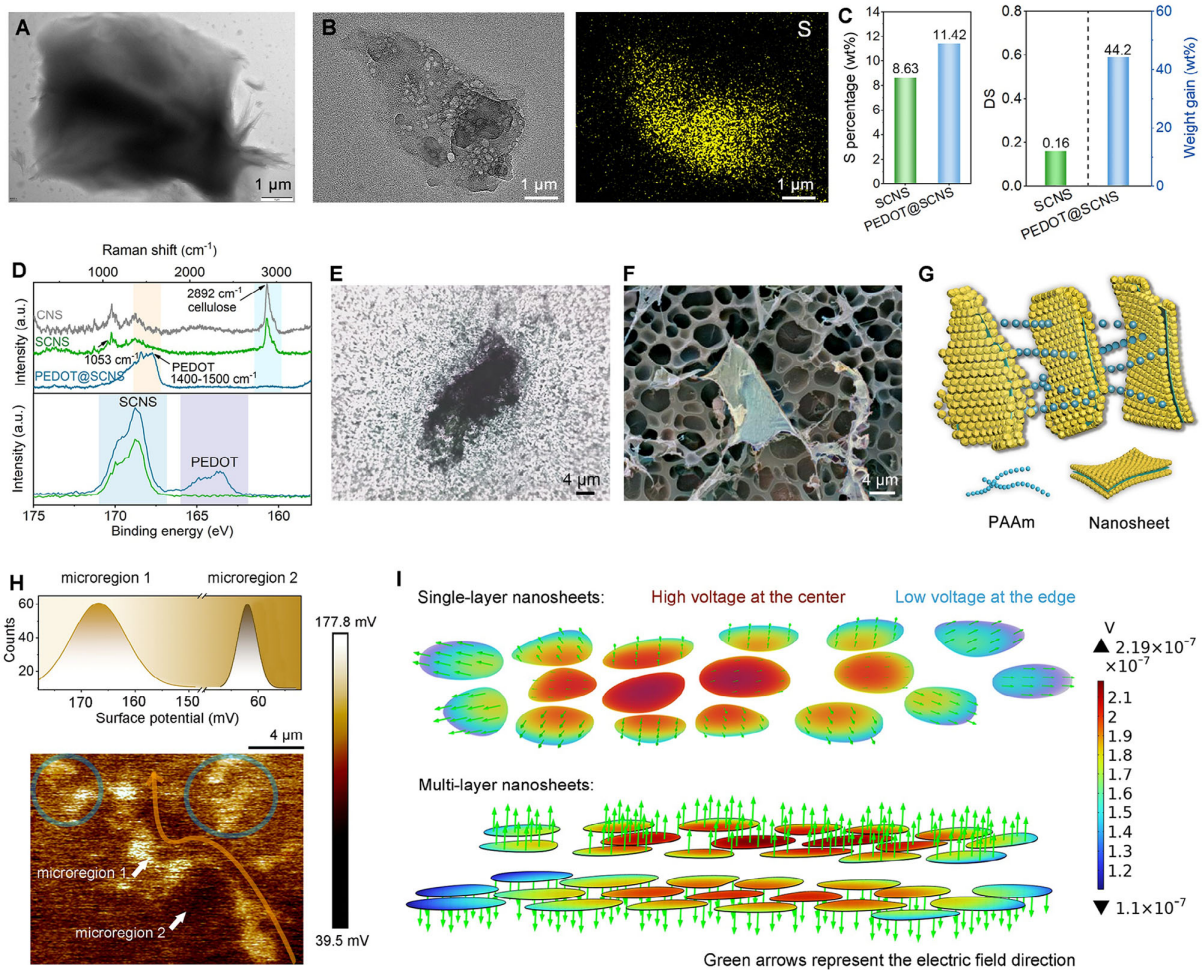
Mechanism of conductive pathway and sensing performance of NSH. TEM images of A) CNS and B) PEDOT@SCNS with EDX mapping of S. C) Mass percentage of S in SCNS and PEDOT@SCNS, DS of SCNS, and weight gain of PEDOT@SCNS. D) Raman, and S 2p XPS of the nanosheets. E) Optical micrograph, F) cryo‐SEM and G) schematic illustration of the microcapacitive hydrogel. H) Surface potential (SP) distribution on NSH using KPFM. The profiles of relative SP values in the upper panel are measured along the white and blue lines in the lower panel. I) Finite element simulation of electric potential distribution in nanosheet‐embedded hydrogels.

CNSs are synthesized from microcrystalline cellulose (MCC) though the dissolution of cellulose in NaOH‐urea eutectics followed by application of strong mechanical shear forces. The resulting structures exhibit various irregular membrane‐like shapes (**Figure** **3**A; Figure S10, Supporting Information). The relatively low transparency and limited wrinkling of these structures indicate that they have a considerable thickness. Following sulfonation and in situ oxidative polymerization of EDOT, the nanosheets retain their 2D morphology while showing improvements in transparency and a decrease in thickness (Figure 3B; Figure S10, Supporting Information). Although some heterogeneities are present, sulfur mapping confirms the successful surface sulfonation and that EDOT has been effectively deposited on the surface of the cellulose nanosheets. As expected, EDOT uniformly polymerizes and adheres to both the top and bottom surfaces of SCNS through electrostatic attraction between the materials. The interaction leads to a formation of a sandwich‐like structure, designated as PEDOT@SCNS, which has an approximate thickness of 170 nm (Figure S11, Supporting Information). The DS of SCNS, measured through elemental analysis, is calculated to be 0.16. After the deposition of PEDOT, there is a significant increase in sulfur content and overall weight of the SCNS. Specifically, the increase in sulfur content is approximately 2.8 wt.%, while the overall weight gain is about 44.2 wt.% (as shown in Figure 3C), suggesting that a substantial amount of PEDOT has been successfully incorporated onto the surface. To further validate the successful surface polymerization of EDOT, characterizations including X‐ray diffraction (XRD), Fourier‐transform infrared spectroscopy (FTIR), Raman spectroscopy, and X‐ray photoelectron spectroscopy (XPS) are conducted (Figure 3D; Figures S12 and S13, Supporting Information).

Both optical and cryo‐scanning electron microscopy (cry‐SEM) imaging confirm the successful integration of conductive nanosheets within the dielectric hydrogel (Figure 3E,F). To validate the hypothesis that these conductive nanosheets function as numerous microelectrodes, forming an array of internal microcapacitors and microresistive pathways in both series and parallel configurations within the dielectric hydrogel (Figure 3G), the spatial potential distribution across the surface of the hydrogel using Kelvin probe force microscopy (KPFM) mapping is examined. The results reveal that the embedded PEDOT@SCNS nanosheets yield different surface potentials compared to the hydrogel matrix (Figure 3H). Specifically, the a mean local potential variations in surface potential are measured at ≈167 mV for the nanosheets and ≈62 mV for the dielectric hydrogel. Thus, we infer that the conductive PEDOT@SCNS nanosheets inherently enhance the local electrical field, creating more favorable conditions for charge storage and transfer. This increase in localized potential further confirms their effectiveness as microelectrodes, facilitating the formation of microcapacitors and continuous conductive networks within the hydrogel. Additionally, electrochemical impedance spectroscopy (EIS) (Figure 1A) was performed, showing capacitive behavior with a well‐defined semi‐circular arc in the Nyquist plot, which is typical of microcapacitors. This further supports the role of PEDOT@SCNS nanosheets as microelectrodes in the formation of microcapacitors within the hydrogel matrix.

The finite element analysis (FEA) indicates that in the single‐layer nanosheet configuration (upper panel), the potential is highest at the center and gradually decreases toward the edges, with the color gradient transitioning from red to blue (Figure 3I). This is because the nanosheets in the center are influenced by the surrounding positively charged nanosheets, leading to a cancellation of forces in the horizontal direction, resulting in a smaller net force. In contrast, nanosheets at the edges are more strongly influenced by the charged nanosheets on one side, leading to a greater net force in the horizontal direction. In comparison, the multi‐layer nanosheet configuration (lower panel) exhibits a more uniform electric field distribution, with interlayer interactions enhancing the overall electric field strength and capacitance effects. The multi‐layer configuration leads to a stronger electric field and more pronounced capacitive effects, thereby improving the sensor's performance.

**Figure 4:**
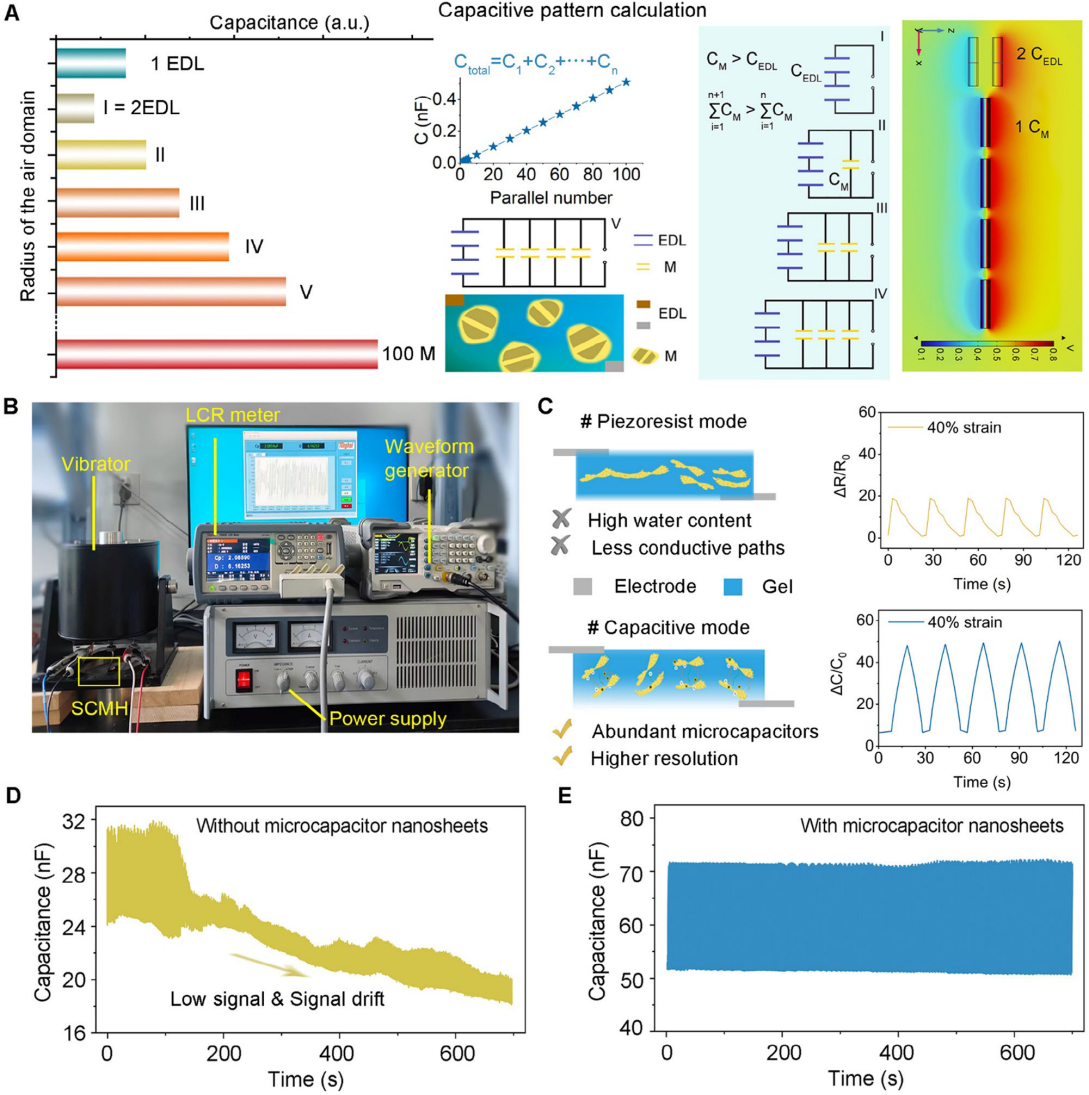
Sensing properties and stability of the dual operational modes. A) Simulated capacitance increments of the hydrogels due to the micro‐capacitor configurations through FEA. PEDOT@SCNS microelectrodes are assumed to be randomly distributed by default within the ultrathin hydrogel, forming internal series‐parallel microcapacitors. B) Experimental setup for evaluating sensing stability under vibration conditions. C) Comparative analysis of sensing performance across two operational modes using NSH containing 2 wt.% nanosheets at a frequency of 0.03 Hz. D,E) Capacitive sensing signals measured by sensors with and without conductive nanosheets.

In addition, the FEA simulation confirms that the simulated capacitance (C) is primarily influenced by microcapacitors in parallel (C_M_) rather than by the two electrical double layers (C_EDL_) formed between the current collectors and the hydrogel (**Figure** **4**A). The study also explores whether the size or the random distribution of the nanosheets within the hydrogel influences signal acquisition and responsiveness. Despite the randomness in distribution and the differences in size, nanosheets naturally create a substantial network of interconnected microcapacitors within the ultrathin hydrogel. FEA calculations provide further insights by demonstrating that in configurations combining series and parallel setups, the C value increases with a higher number of microcapacitors, regardless of their distribution patterns. Moreover, FEA calculations show that the capacitance increases nearly linearly with the number of nanosheets arranged in parallel, highlighting the dominant role of nanosheet‐derived microcapacitor arrays in enhancing signal strength and responsiveness, regardless of spatial configuration.

Further, the stability of sensors with and without embedded microelectrodes under harsh mechanical conditions (3 kPa cyclic loading; Figure 4B) is compared. It is hypothesized that when nanosheets are distributed randomly at lower concentrations (1‒4 wt.%) within the hydrogel matrix, there are more microcapacitors formed relative to the conductive pathways, leading to enhanced sensor's response in capacitive sensing mode than in piezoresistive mode. Testing results confirm that, at these lower concentrations, a greater number of PEDOT@SCNS nanosheets function effectively as microcapacitor electrodes. As a result, the capacitance of the sensor varies more significantly with strain than resistance does (Figure 4C). Additionally, the NSH sensor demonstrates significantly more stable signal output when subjected to vibrations compared to the hydrogel without nanosheet‐based microcapacitors (Figure 4D,E). Furthermore, hydrogels with higher concentrations of nanosheets show a marked improvement in both sensing signal intensity and stability (Figure S14, Supporting Information). In contrast, hydrogels with lower concentration of nanosheets, particularly those that lack nanosheets altogether, exhibit weaker signals and considerable baseline drift when subjected to the same forces.

### 2.4 Strain Sensing Performances

**Figure 5:**
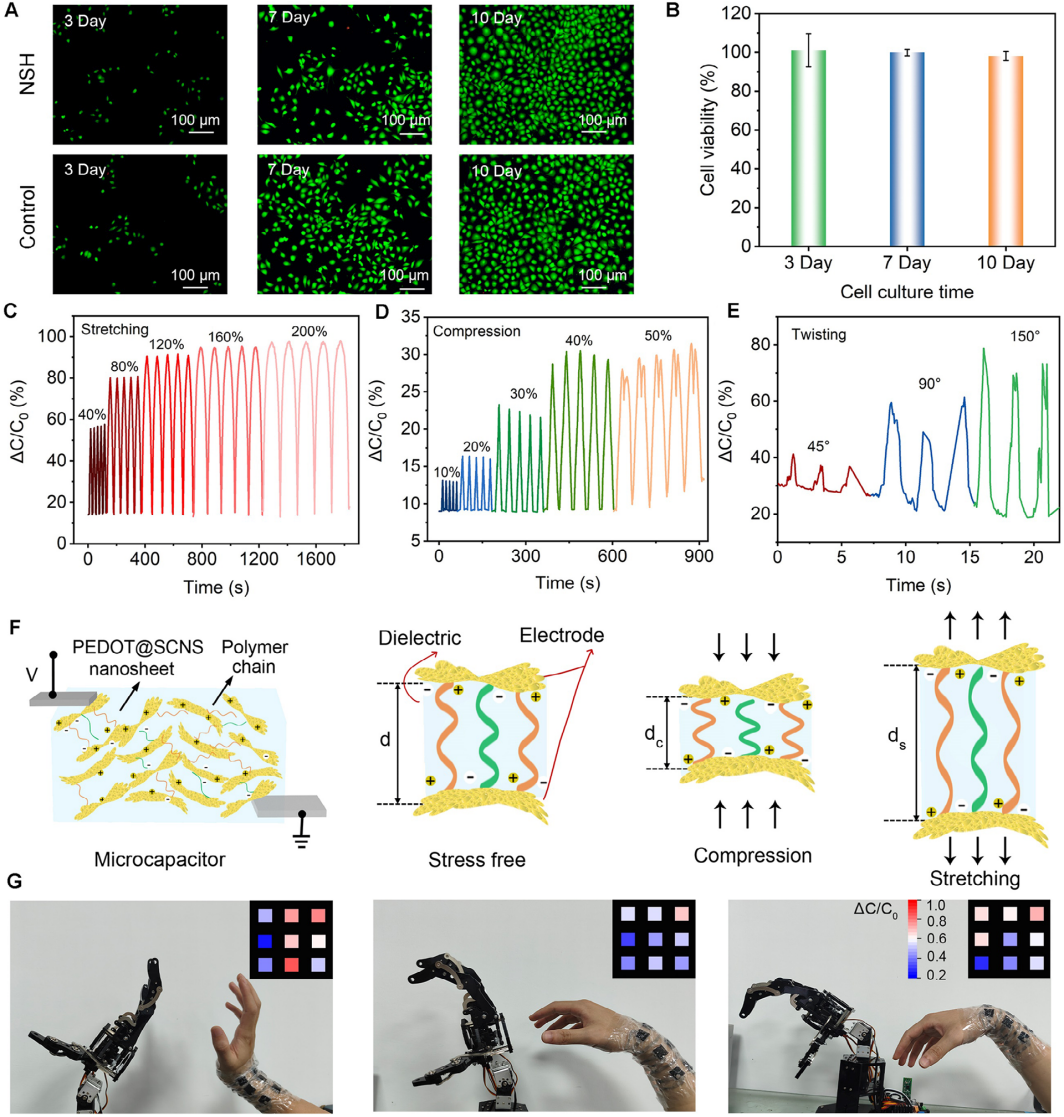
Physiological performances of the 1.8 M NSH sensor. A) Cell viability reflected by staining the adherent living cells. B) Data of CCK‐8 assay for HUVECs after culturing with or without hydrogels. Responsiveness toward (C) stretching, (D) compression, and (E) twisting. F) Mechanosensing sensing mechanism based on PEDOT@SCNS. G) A real‐time multichannel control model for human‐machine interface in operating a mechanical hand.

Given that the hydrogel comes into direct contact with the skin, it is essential to evaluate its safety and biocompatibility prior to assessing its sensing performance. To evaluate its long‐term cytocompatibility, we conducted cytotoxicity tests over a period of 10 days. In these experiments, human umbilical vein endothelial cells (HUVECs) were cultured in wells with and without the hydrogel for 3, 7, and 10 days. After the culture period, the living cells were stained bright green, indicating a comparable number of viable cells in both the hydrogel and control groups, even after prolonged exposure. This demonstrates that the hydrogel does not induce significant cytotoxic effects over extended periods, further confirming its excellent biocompatibility. **Figure** **5**A provides a visual representation of the fluorescence staining results, showing sustained cell adhesion and proliferation in the hydrogel‐exposed group. The results from the CCK‐8 assay further confirm the hydrogel's biocompatibility, as cell viability remained consistently high over 3, 7, and 10 days, supporting the findings from the cell viability staining (Figure 5B).

Due to the inclusion of microelectrodes and high compliance, our NSH holds promising potential for applications as motion sensors in wearable electronics. For strain sensing, the responsiveness of the hydrogel‐based sensor is evaluated using the relative change of capacitance, expressed as ΔC/C_0_ = (C‐C_0_)/C_0_ (where C_0_ is the initial capacitance and C is the real‐time capacitance after deformation). The 1.8 m hydrogel‐based strain sensor demonstrates excellent and stable sensing capability in two distinct scenarios: stretching‐releasing within a strain range of up to 200% and compressing‐releasing within a strain range of up to 50% (Figure 5C,D). The sensor's gauge factors, which indicate the sensitivity to strain, are calculated as follows: 1.65 for strains up to a 40%; 1.12 for strains between 40–80%, and 0.69 between 80 and 200%. Additionally, the sensor's sensitivity to compressive stress reaches 1.74 kPa^−1^ within an 8 kPa range (Figure S15, Supporting Information). This performance ranks among the top in most previously reported literature. These results highlight the sensor's adaptability and precise responsiveness across a range of strain levels.

Additionally, the NSH sensor exhibits excellent durability and reliability, maintaining a constant amplitude and signal waveform over 600 sequential cycles at 100% strain (Figure S16, Supporting Information). The sensor exhibits minimal signal drift and consistent capacitance response even after 2000 compression cycles (Figure S17, Supporting Information). Furthermore, the sensor demonstrates stable performance under electrical stimuli, with consistent signal and minimal drift even after prolonged electrical stimulation (Figure S18, Supporting Information). These results highlight the sensor's exceptional durability and reliability under both mechanical electrical stimuli. The measured response time is influenced by the data extraction frequency of the equipment, typically reported in milliseconds. Moreover, when suffering twisting deformation, the values of ΔC/C_0_ also steadily and rapidly increase, reaching 80% at a twisting angle of 150° (Figure 5E), suggesting the hydrogel's strong capability to sense various types of deformation.

The responsive capacity of NSH originates from the spatial adjustments between the PEDOT@SCNS conductive nanosheets which form a network of microscale‐width conductive layers dispersed throughout the hydrogel network, upon deformation (Figure 5F). Each pair of conductive nanosheets, separated by the hydrogel, acts as an individual microcapacitor. The abundance of these nanosheets distributed throughout the hydrogel creates a vast network of microcapacitors. Namely, when the hydrogel undergoes **compression,** the conductive layers come closer, increasing capacitance; during **stretching,** the layers separate, decreasing capacitance. This dense configuration significantly enhances the sensor's responsiveness and sensitivity. The bulk capacitance in the hydrogel is achieved through a series‐parallel network of microcapacitors. This setup amplifies the sensor's signal response, resulting in higher sensitivity and better detection of subtle deformations.

Our wearable multichannel human‐machine interfaces based on NSH sensors can be applied in various applications, for example, operating a mechanical hand (Figure 5G). The hydrogel sensors detect capacitive changes caused by hand motions in real‐time, which are then converted into digital signals. These signals are subsequently transmitted in real‐time to the mechanical hand for hand gesture recognition (Movie S1, Supporting Information). The results demonstrate that the mechanical hand can accurately and promptly replicate the motions of human fingers (Figure S19, Supporting Information) and wrists (Figure 5G).

### 2.5 Electrophysiology Sensing Performances

#### 2.5.1 Advanced EMG and Facial Nerve Monitoring

**Figure 6:**
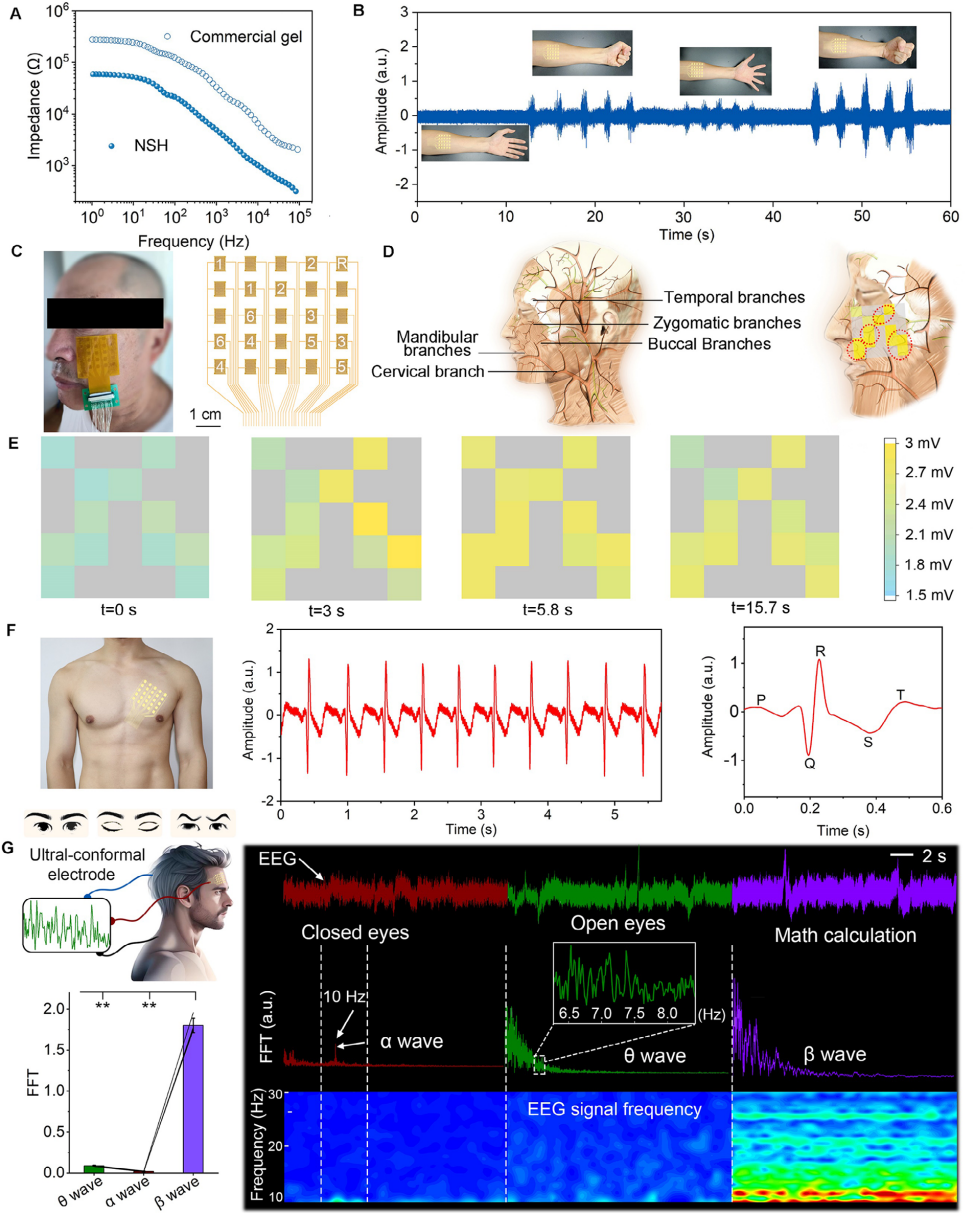
High‐fidelity recording of electrophysiological signals using NSH electrodes. A) Skin interfacial impedances of the NSH and commercial gel electrodes. B) Electromyography (EMG) signals recorded from the arm. C) Photos, circuits and D) diagram of facial nerves for facial EMG measurements. E) Spatiotemporal distribution array of facial EMG signals. F) Electrocardiography (ECG) signals captured and magnified view of a cardiac cycle. G) Electroencephalography (EEG) rhythms recorded during different mental states. Frequency distributions of the EEG signals processed using FFT and time‐frequency spectrograms of the EEG signals within the alpha and beta bands (10‐38 Hz) during three mental states (Left: closed eyes; Middle: open eyes; Right: math calculation) are presented. Statistical details for one‐way repeated measures ANOVA (*p* < 0.001) are shown in Table S2 (Supporting Information).

The introduction of conductive nanosheets facilitates a tight interface contact between the skin and the electrode, enhances the electrical conductivity of the electrode, and reduces the resistance to electrical flow (Figure S20, Supporting Information). Additionally, impedance and conductivity analyses demonstrate that lower AAm concentrations facilitate ion transport, reducing impedance while maintaining stable bulk conductivity. As a result, the interfacial impedance between human forearm skin and the NSH electrode is significantly lower compared to that of traditional electrodes, such as the commercial Ag/AgCl gel electrode (**Figure** **6**A). This lower impedance leads to better signal quality and more accurate readings.

When the biocompatible, low‐skin‐impedance NSH is applied to the musculus biceps brachii of a volunteer, it is integrated with a multichannel physiological signal acquisition and processing system. This integration allows for the real‐time detection of EMG signals generated by muscle contractions. The resulting EMG signals produce output voltages of ≈1.1 mV (Figure S21, Supporting Information). The system can easily and accurately identify characteristic EMG voltages that correspond to different muscle states or actions, such as relaxation, loose grip, tightening, and firm grip (Figure 6B).

Facial nerve dysfunction is a relatively common condition that affects the facial nerve (cranial nerve VII). To monitor the electrical activity associated with facial nerve function, NSH electrophysiological epidermal electrode arrays on multichannel interdigitated electrode current collectors arranged in a 5 × 5 configuration on a flexible **polyimide (PI)** substrate measuring 5 cm × 5 cm are developed as shown in Figure 6C. The NSH electrode arrays enable spatiotemporal monitoring of facial EMG using our NSH electrode arrays which involves recording the electrical activity generated by facial muscles. The results demonstrate that the electrode arrays clearly capture variations in the frequency and intensity of nerve impulse irregularities (Figure 6D,E). Furthermore, to evaluate real‐world applicability, we conducted dynamic condition testing, including muscle activity monitoring during walking and running. The NSH arrays demonstrated excellent signal resolution and mechanical stability during these tests, reinforcing their potential for practical wearable applications. Figure S22 (Supporting Information) illustrates spatiotemporal distribution array of facial EMG signals during walking and running states (Figure S22, Supporting Information). The ability of the NSH technology to monitor facial EMG signals holds significant promise for early diagnosis, treatment efficacy evaluation, recovery progress monitoring, and complication detection.

#### 2.5.2 Advanced ECG and Brain Activity Monitoring

The ECG spectrum obtained from a healthy individual using a simplified three‐electrode method displays well‐defined and highly accurate peaks corresponding to the key components of the cardiac cycle: P wave, QRS complex, and T wave (Figure 6F). These distinct waveforms reflect the synchronized depolarization and repolarization processes of the atria and ventricles, enabling precise real‐time heart rate monitoring and accurate diagnosis of a wide range of cardiac conditions, thus allowing for comprehensive analysis and identification of potential heart diseases. In addition, the high adaptability and adhesiveness of our hydrogel prevent the occurrence and expansion of interfacial gaps between the electrode and the skin surface while exercising, leading to extremely stable sensing and minimal motion artifacts even during dynamic conditions (Figure S23, Supporting Information).

When compared to the commercial Ag/AgCl hydrogel electrode, the signal‐to‐noise ratio (SNR) values recorded by the novel hydrogel (e.g., 22.9 dB for EMG in static state and 20.6 dB for EMG while running) are significantly higher than those recorded by Ag/AgCl electrodes (10.8 dB for EMG in static state and 5.7 dB for EMG while running), especially in dynamic conditions. The corresponding root‐mean‐square (RMS) noise values for NSH are 9.7 µV while sitting and 11.5 µV while running, which are also much lower and superior to those of the commercial electrode, at 33.5 and 48.7 µV, respectively (Figure 1A). It's worth noting that Dr. Bao's group has demonstrated an improved SNR of 33.7 dB by etching a hydrogel micropillar surface topology.^[^ ^32^ ^]^ This suggests that there is potential for further enhancing the SNR of the novel hydrogel by employing similar surface modifications, if necessary. Nonetheless, the sensing resolution, durability, and stability of the novel hydrogel ranks among the highest for state‐of‐the‐art electrophysiological hydrogel electrodes reported in the literature (Table S3, Supporting Information).^[^ ^33^ ^]^

These on‐skin hydrogel electrodes can be applied to monitor brain wave activities associated with different mental states via real‐time EEG recording (Figure 6G). To identify and analyze the brain waves, Fast Fourier Transform (FFT) tool is used to convert the EEG signal from the time domain to the frequency domain. During the testing process, participants are instructed to first close their eyes, enter a relaxed state, then open their eyes while maintaining a state of thoughtlessness, and finally perform complex math calculations. When participants closed their eyes and entered a relaxed state, the EEG recordings show presence of alpha (α) waves (≈10 Hz); while the open‐eye state is marked by the appearance of theta (θ) waves (∼6.5 Hz). Active thinking process elicits high‐frequency, low‐amplitude beta (β) waves (12–40 Hz).

To quantify the difference in FFT spectral profiles obtained for different mental states (such as relaxation, thoughtlessness, and active problem‐solving), the mean normalized power across EEG frequencies ranging from 1 to 40 Hz is measured, and one‐way repeated measures ANOVA is employed to determine whether the mean power values varied significantly across the mental states (Figure 6G). The data are normalized to allow direct comparison of mean power levels for each brain wave type (α, θ, and β), over 15‐s intervals in each mental state. The time‐frequency spectrograms of the recorded EEG signals reveal significant differences in signal intensities across these three mental states. Notably, the EEG signals recorded by our NSH electrodes exhibit spectral characteristics and signal intensity distributions that are highly consistent with those reported in previous studies.^[^ ^3^, ^34^ ^]^ Continuous monitoring of cerebral activities through high‐fidelity, real‐time EEG recording is of paramount significance in investigating cognitive behaviors and provides valuable insights into diverse neurological disorders and human fatigue.

## 3 Conclusion

This study enables the development of high‐quality, long‐term stable bioelectronic interfaces that are resistant to external interference. The super‐compliant hydrogel forms bulk microcapacitor and microresistance junctions through numerous conductive nanosheets connected in series and parallel, leading to significant signal amplification, enhanced sensitivity, and reduced motion artifacts. To better understand the electrical behavior of the NSH sensor, we employed the parallel capacitor model in FEA simulations to describe its capacitive response. Besides, alternatives such as the Debye or RC transmission line models could provide further insights into frequency‐dependent dielectric properties and charge transport dynamics, offering potential frameworks for future studies. Overall, the performance of our NSH is comparable to that of state‐of‐the‐art materials reported by leading research teams, with high gauge factor, superior SNR, and excellent biocompatibility. We demonstrate the successful application of these nanosheet‐based, super‐compliant hydrogel patches as mechanosensors for monitoring complex motions and as electrodes for recording physiological signals. With its enhanced mechanical, flowability, electrical, and assembly properties, we anticipate that this novel dual‐operating‐mode hydrogel sensor will enable wide‐ranging applications in next‐generation flexible epidermal electronics.

## 4 Experimental Section

### Materials

Microcrystalline cellulose (MCC), sodium hydroxide (NaOH), urea, sulfamic acid (99.5%), N,N‐Dimethylformamide (DMF, 99.5%), 3,4‐Ethylenedioxythiophene (EDOT, 99%), iron (III) chloride (99%), ammonium persulfate (APS, 98%), anhydrous ferric chloride (FeCl_3_, 98%), carboxymethylcellulose (CMC, degree of substitution (DS) 0.7), acrylamide (AAm), N,N'‐methylenebisacrylamide (BIS), and N,N,N′,N′‐tetramethylethylethylenediamine (TEMED, 99%) were purchased from Aladdin Reagent (Shanghai, China). Commercial gel electrode (Ag/AgCl) was purchased from Chengdu Instrument Factory (Sichuan, China). The 3D‐printed patterned substrates were purchased from Suzhou Infinite 3D Technology Industry Co., Ltd (Jiangsu, China). Porcine skin was purchased from Jingdong (Beijing, China) and stored in PBS buffer (0.01 M, pH 7.4) before use. Biological materials including fetal bovine serum (FBS), Dulbecco's Modified Eagle Medium (DMEM, Hyclon), cell culture dishes, CCK‐8 cell counting kit were purchased from Shanghai Yisheng Biotechnology Co., Ltd., China.

### Synthesis of CNS

MCC was dissolved in the 7 wt.% NaOH/12.0 wt.% urea aqueous solution by repeated freeze‐thaw treatment (3 cycles).^[^ ^35^ ^]^ The freezing temperature was ‐18 °C and the thawing process was carried out at room temperature (23 °C) under vigorous stirring. The resultant MCC solution (100 mL) was poured into 1 L deionized (DI) water followed by high‐speed dispersion with a high‐speed disperser (IKA T25, Staufen, Germany) at a speed of 10 000 rpm for 20 min to produce CNSs. CNSs were then washed via dialysis and freeze‐dried using a LABCONCO, Free Zone 6 instrument (Kansas, USA).

### Synthesis of SCNS

The freeze‐dried CNSs (2 g) were dispersed in 50 mL sulfamic acid/DMF (5 wt.%) and heated at 80 °C for 2 h while stirring. The resulting SCNS suspension was dialyzed with DI water to remove unreacted reagents and then concentrated at 85 °C under atmospheric pressure to reach a concentration of ≈ 0.5 wt.%.

### Synthesis of PEDOT@SCNS

For in‐situ PEDOT polymerization on the surface of SCNSs, 0.05 g EDOT was added to 10 mL as‐prepared SCNS suspension. After stirring for EDOT dissolution, APS (200 wt.% relative to EDOT) and FeCl_3_ (6 wt.% relative to EDOT) were added. The mixture was stirred at room temperature for 24 h to obtain PEDOT@SCNS.

### Preparation of NSH

CMC (0.8 wt.%) was dissolved in 10 mL DI water followed by the addition of the prepared PEDOT@SCNS dispersion (0.14, 0.28, 0.43, and 0.57 mL) under stirring for 10 min. Then, AAm (1.8, 2, 2.5, and 3 m), BIS (0.3 wt.% relative to AAm), APS (0.8 wt.% relative to AAm), and TEMED (10 µL) were dissolved in the mixture under stirring for 30 min. The solution was poured into a custom‐made flat‐bottomed glass mold and vacuumed to remove bubbles and oxygen followed by heating to 55 °C for 30 min, for gelation. The hydrogels with additional macromolecules that allow for the incorporation of different functional groups into the structure of hydrogels were synthesized using a straightforward mixing method based on the 1.8 m hydrogel formulation (Table S1, Supporting Information).

### Micromorphology and Composition Characterization

The microstructure of CNSs, SCNSs, PEDOT@SCNSs, and hydrogels was characterized by scanning electron microscope (SEM) SU8010 (Hitachi, Japan) equipped with a Bruker XFlash 6160 energy‐dispersive spectrometer (EDS, BRUKER XFlash 6160, Germany) and Transmission electron microscope (TEM) Tecnai G2 F30 S‐Twin (FEI, Hillsboro, USA). Atomic force microscopy (AFM) investigation was carried out on a Bruker Dension Icon (Bruker Co., Ltd., Zurich, Switzerland). Fourier‐transform infrared spectroscopy (FTIR) was performed on a Nicolet IS10 instrument (Thermo Fisher, Waltham, USA) in the range of 400–4000 cm^−l^. Crystal phase changes were characterized by powder X‐ray diffractometry (XRD) on a D8 ADVANCE instrument (Bruker, Germany) using Cu Kα radiation (λ = 1.54 Å) generated at a voltage of 40 kV, and a current of 100 mA, at a scanning rate of 4 °min^−1^, from 10° 2θ to 90° 2θ. X‐ray photoelectron spectroscopy (XPS) was conducted with the ThermoFisher K‐Alpha XPS system (Waltham, USA). Raman spectroscopy was performed using a Renishaw RM‐2000 Laser Raman Spectrometer (Gloucestershire, UK) using a 532 nm laser as the excitation source. 2D Raman mapping was performed on a Renishaw inVia Raman microscope (Gloucestershire, UK) in a backscattering configuration.

### Mechanical and Compliant Properties

A 50 mm length, 10 mm width, and 2 mm thickness hydrogel were taken to assay the tensile curves using a universal testing machine (Instron 5967, Boston, USA) at a stretching speed of 10 mm min^−1^ using a 200 N load cell. Rheological characterization of the hydrogels was carried out by using an ARES‐G2 rheometer (TA Instruments, New Castle, USA). The frequency sweep mode was performed over a frequency range of 0.02 – 100 rad s^−1^, keeping the strain constant at 0.5%, at 25 °C.

### Adhesiveness of Hydrogels

The adhesive properties of the hydrogels were investigated by the standard lap‐shear test and conducted on a universal testing machine (Instron 5967, Boston, USA) equipped with a 10 kN load‐cell with a constant tensile speed of 10 mm min^−1^. Samples for lap‐shear testing were prepared by adhering two pieces of the substrate using thin square hydrogels with a 20 mm length, 20 mm width, and 0.5 mm thickness. Stereomicroscopy was carried out using a Keyence VHX‐1000 digital microscope (Osaka, Japan).

### Electrical Properties

Electrochemical impedance spectroscopy (EIS) measurements were carried out by using an electrochemical workstation (CHI760, CH Instrument Inc., China) with a sweep range from 100 kHz to 1 Hz at an open‐circuit potential of 0.1 V in a three‐electrode electrochemical cell. The reference electrode used was an Ag/AgCl/KCl (3M) single‐junction electrode (Model 6.0733.100, Metrohm), with a Pt foil serving as the counter electrode and 1 m H_2_SO_4_ solution as the electrolyte. The working electrode was the NSH on the electrode clip. The contact impedance of electrode‐skin was measured in the range from 1 Hz to 100 kHz with the applied voltage of 0.01 V. The electrical conductivity of hydrogel samples was tested by a four‐point probe (Guangzhou 4 probes tech RTS‐8 type, China).

### Mechanosensing Capacity

SP images were measured using the single‐pass and amplitude‐modulated Kelvin probe force microscope (Single‐pass AM‐KPFM) on a commercial AFM (Cypher S, Asylum Research, USA) with a conductive tip coated with Pt/Ir (SCM‐PIT‐V2, Bruker). During the measurement, a direct voltage (DC voltage) and an alternating voltage (AC voltage) were applied to the sample or the tip. The DC voltage was used to nullify the contact potential difference (CPD) between the sample and the tip, while the AC voltage was applied to extract the immediate electric potential signal once the CPD was nullified by the DC voltage. The capacitance outputs of the hydrogels were tested by a digital bridge (Tonghui TH2830, China). For the motion sensing for manipulator controlling, the capacitance versus deformation was recorded and analyzed with Keil u5 (Keil Software Inc., San Francisco, USA).^[^ ^36^ ^]^ Signal processing and command generation were performed on a signal transfer module in the above controller. Finite element analysis (FEA) was employed to simulate the effect of microcapacitor configurations on the sensing performance of hydrogels using COMSOL Multiphysics.

### In Vitro Biocompatibility Tests

The cytocompatibility of the hydrogels was assessed using human umbilical vein endothelial cells (HUVECs). Briefly, HUVECs (2×10^3^ cells per well) were seeded into 96‐well plates and incubated for 12 h. Subsequently, hydrogel samples (6 mm × 6 mm × 2 mm) were added for co‐culture. After 3, 7, and 10 days of incubation, 100 µL of CCK‐8 working solution (10% in DMEM) was added to each well and further incubated for 1 h. The absorbance at 450 nm was then measured using a microplate reader (Thermo, Multiskan Go, USA). In addition, live/dead staining was performed using calcein‐AM and propidium iodide, and cell viability was observed under an inverted fluorescence microscope (Nikon ECLIPSE Ti).

### Electroencephalography Signal Recording

The electromyography (EMG), electrocardiography (ECG or EKG), and electroencephalography (EEG) data were recorded by measuring the signals through the hydrogel electrodes using a multichannel physiological signal acquisition and process system (RM620C, Chengdu Instrument Factory, China). In particular, facial EMG signals were acquired using an EMG operational module, enabling real‐time monitoring and analysis of muscle activity (Figure S24, Supporting Information). The experiments were performed in compliance with the protocol approved by the ethical committee at Zhejiang A & F University (approval number ZAFUAC202452, Hangzhou, China). Informed consent was obtained from the volunteers for recording of the electrophysiological signals. The root‐mean‐squared (RMS) noise of ECG signals and the signal‐to‐noise ratio (SNR) of electrophysiological signals in different motion states were calculated using the following Equations (1) and (2), respectively:^[^ ^6^, ^37^ ^]^

(1) $$\;X_{\mathrm{RMS}\;}=\sqrt{\frac{\sum_{i=1}^{n}X_{i}^{2}}{N}}=\sqrt{\frac{X_{1}^{2}+X_{2\;}^{2}+\text{…}+X_{n}^{2}}{N}}$$

(2) $$\mathrm{SN}R_{\mathrm{dB}}=10\;\mathrm{lo}g_{10}{\left(\frac{A_{\mathrm{signal}}}{A_{\mathrm{noise}}}\right)}^{2}=20\;\mathrm{lo}g_{10}\left(\frac{A_{\mathrm{signal}}}{A_{\mathrm{noise}}}\right)$$

All EEG data (5 parallel tests for each EEG state) were compared using one‐way ANOVA after FFT transformation.

### Statistical Analysis

All data are presented as mean ± standard deviation (SD). The sample size (n) for each dataset is specified in the corresponding figure legends. For multiple group comparisons, one‐way analysis of variance (ANOVA) followed by Tukey's post‐hoc test was applied to determine statistical significance. A *p*‐value less than 0.05 was considered statistically significant (*p* < 0.05, p < 0.01, *p* < 0.001). All statistical analyses were performed using SPSS Statistics and Excel 2019, Origin 2018 was used for data organization and plotting.

## Conflict of Interest

The authors declare the following financial interests: J. L. Shamshina has partial ownership of Chitalyst, LLC and Mirus Boletus, LLC.

## Supporting information



Supporting Information

Supplemental Movie 1

## Data Availability

The data that support the findings of this study are available from the corresponding author upon reasonable request.
