# Supplementary material for: Dual‐Working‐Pattern Nanosheet‐Based Hydrogel Sensors for Constructing Human‐Machine and Physiological‐Electric Interfaces
Source: Adv Sci (Weinh). 2025 Jun 29;12(33):e14301. doi: 10.1002/advs.202414301 (PMC12412529; doi:10.1002/advs.202414301)
Supplement: Supplementary file 1 — Supporting Information [file ADVS-12-e14301-s002.docx]

Supporting Information

**Dual-Working-Pattern Nanosheet-Based Hydrogel Sensors for Constructing Human-Machine and Physiological-Electric Interfaces**

Shitao Shi,^1^ Yuanyuan Wang,^1^ Zewei Ye,^1^ Hongxia Xie,^1^ Chencong Liu,^1^ Jiaqi Liao,^1^ Dawei Zhao,^2^ Qingfeng Sun,^1,^* Julia L. Shamshina,^3,4,^* Xiaoping Shen^1,^*

1. College of Chemistry and Materials Engineering, Zhejiang A&F University, Hangzhou, 311300, China.
2. Key Laboratory on Resources Chemicals and Materials of Ministry of Education, Shenyang University of Chemical Technology, Shenyang, China.
3. Fiber and Biopolymer Research Institute, Department of Plant and Soil Science, Texas Tech University, Texas, 79409, USA.’
4. Department of Chemistry and Biochemistry, Texas Tech University, Texas, 79409, USA.

*Corresponding to: QS: qfsun@zafu.edu.cn; JLS: jshamshi@ttu.edu; XS: xpshen@zafu.edu.cn

Supplementary Text

The zeta potential value of the suspension precursor of the hydrogel is *ca.* -20 mV (**Figure S1 A**), an indicator of medium dispersing stability. Upon the incorporation of CMC, the zeta potential remarkably decreases to -50 mV, which can be explained by the TEM observation that PEDOT@SCNSs uniformly and firmly stick to the CMC macromolecules (**Figure S1 B**) through electrostatic interactions (**Figure S2**).

While MCC exhibits diffraction peaks belonging to cellulose I at 15.2°, 22.3° and 34.5° 2θ corresponding to (110), (002), and (004) planes, respectively, regenerated CNSs display typical diffraction of cellulose II at 11.8°, 19.8° and 21.7° 2θ related to the (10), (110), and (020) crystal planes,^[1]^ respectively (Figure S12 A, Supporting Information). After sulfonation, the crystalline structure of SCNS remains virtually unchanged. Notably, in PEDOT@SCNS, the prominent characteristic diffraction peak emerging at 26.1° can be assigned to the (020) plane of an orthorhombic unit cell of PEDOT, indicating face-to-face stacking.^[2]^ The new small peak at 17.7° 2θ may correspond to the (200) direction, showing PEDOT edge stacking.^[3]^ Alternatively, the peaks observed at 17.7° and 23.2° might result from the first-order and second-order diffractions originating from the intense 26.1° peak.^[4]^

As shown in FTIR spectra, the constituents of regenerated cellulose are identical to those of the original cellulose (Figure S12 B, Supporting Information). After sulfonation, the peaks of SCNS at 855 cm^-1^ and 1221 cm^-1^ correspond to the stretching vibrations of C–O–S and O=S=O bonds, respectively. For PEDOT@SCNS, the characteristic C=C stretching at 1532 cm^-1^ originates from the ethylenedioxy bond in the PEDOT structure, and the vibration bands of C–C stretching at 1211 cm^-1^ are associated with the quinoidal structure of the thiophene ring. The characteristic C–S bond stretching is observed at 945 and 678 cm^-1^ originating from the ethylenedioxy ring.^[3, 5]^

In comparison with the pristine CNSs, the Raman spectrum of SCNS shows an additional peak located at 1053 cm^-1^, which is attributed to the symmetric stretching vibration of υ_sym_(O=S=O) sulfate groups (Figure 3D).^[6]^ After further PEDOT assembly, new strong peaks appear in the range of 1400-1500 cm^-1^, corresponding to the stretching vibration of double bond C_α_=C_β_ on the five-member thiophene ring of PEDOT, while the characteristic peak of cellulose located around 2892 cm^-1^ decreases.^[7]^ The X-ray photoelectron spectroscopy (XPS) spectra of SCNS and PEDOT@SCNS exhibit peaks at approximately 532.4, 401.4, 285.4, and 169.5 eV, corresponding to O 1s, N 1s, C 1s, and S 2p, respectively, and indicating the presence of O, N, C, and S elements in both types of nanosheets (Figure S13, Supporting Information). Unlike CNS, PEDOT@SCNS exhibits new energy peaks between 163 and 166 eV, which are ascribed to the thiophene rings on the PEDOT chain (Figure 3D).


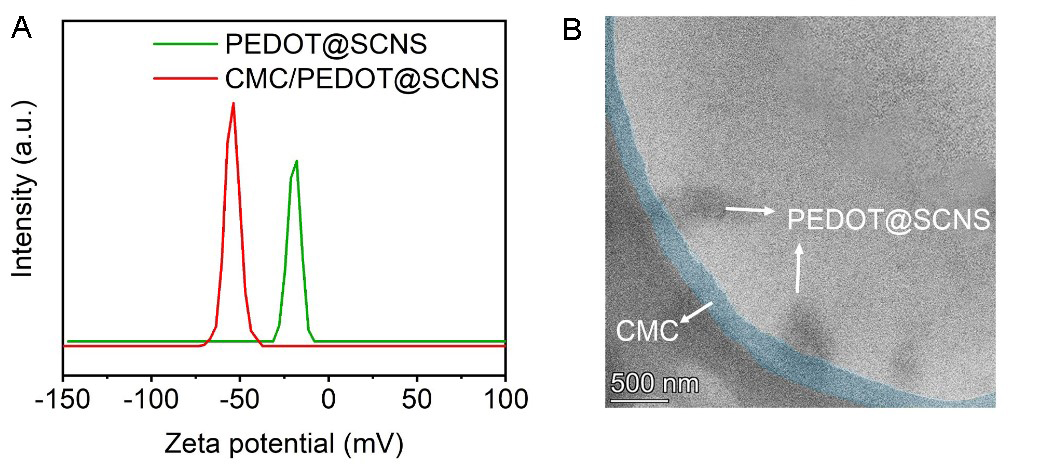


Figure S1. Dispersibility of PEDOT@SCNS suspensions reflected by A) Zeta potential and B) TEM image. The addition of CMC further improves the dispersion stability.


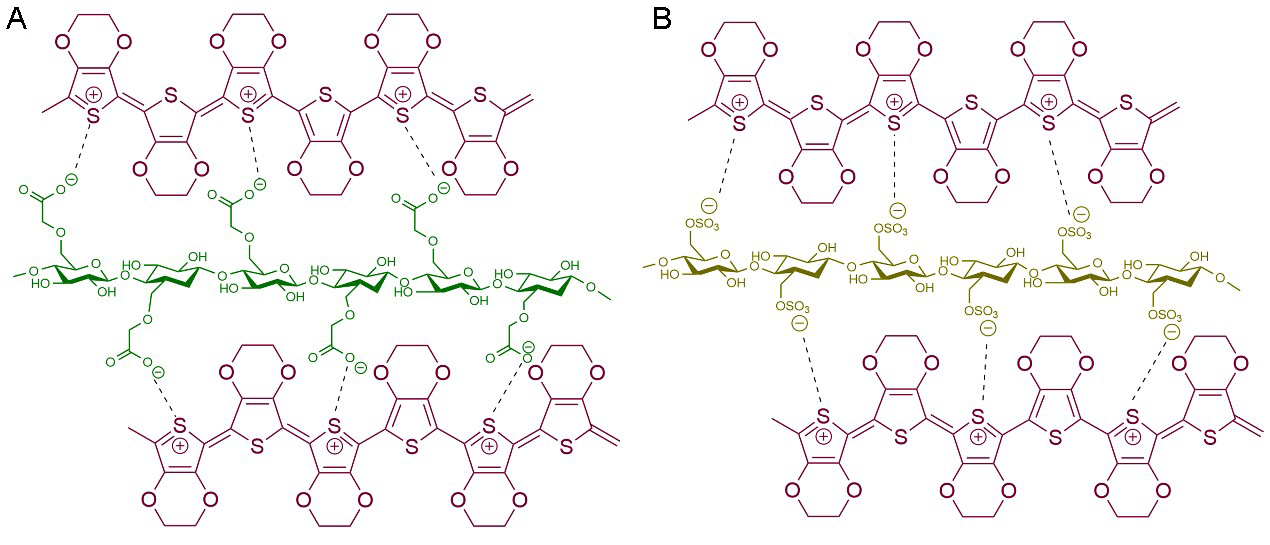


Figure S2. A schematic representation of interactions between positively charged PEDOT and negatively charged CMC (A) and SCNSs (B).


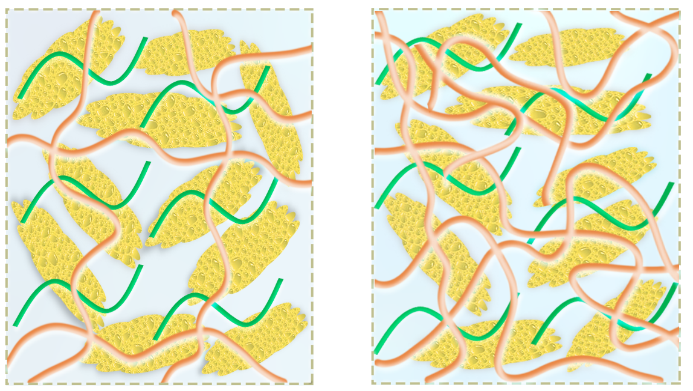


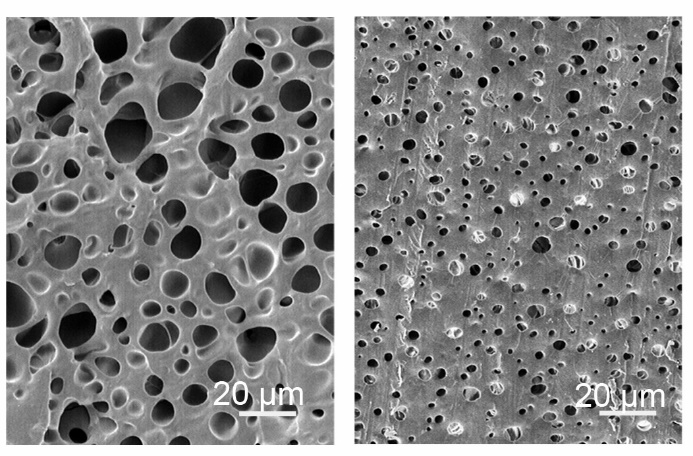


Figure S3. Microstructure of the hydrogel controlled by the concentration of PAAm (Left: 2M hydrogel, Right: 3M hydrogel).


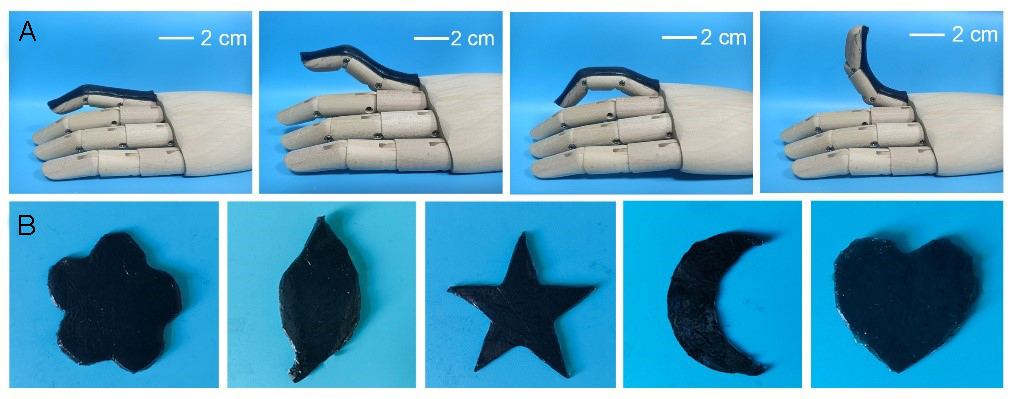


Figure S4. A) Dynamic adaptation of the hydrogel to the highly nonlinear surface of a prosthetic finger, allowing for accommodation of finger movements. B) Manipulation and adhering of the hydrogels to substrates of different shapes.





**Figure S5.** Loss factor tan δ values of SCMH with different AAm concentrations.


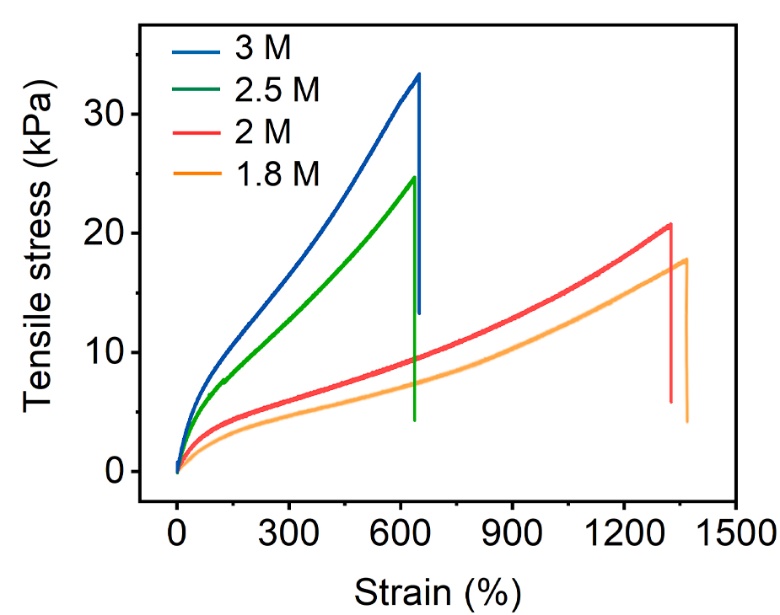


**Figure S6.** Tensile properties of SCMH samples with different AAm concentrations.


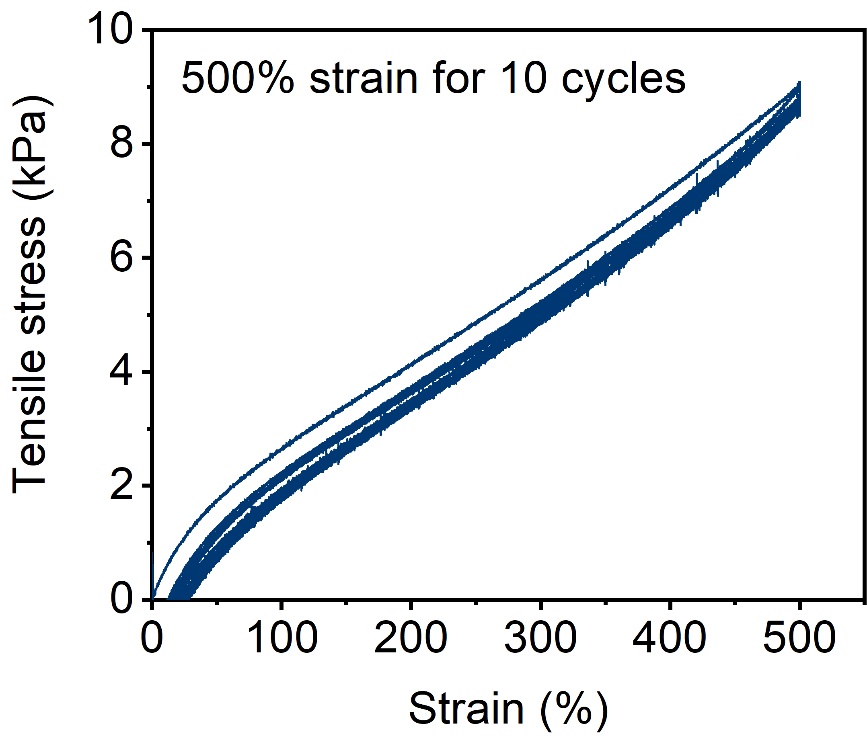


**Figure S7.** Tensile loading-unloading curves of the 1.8M hydrogel.


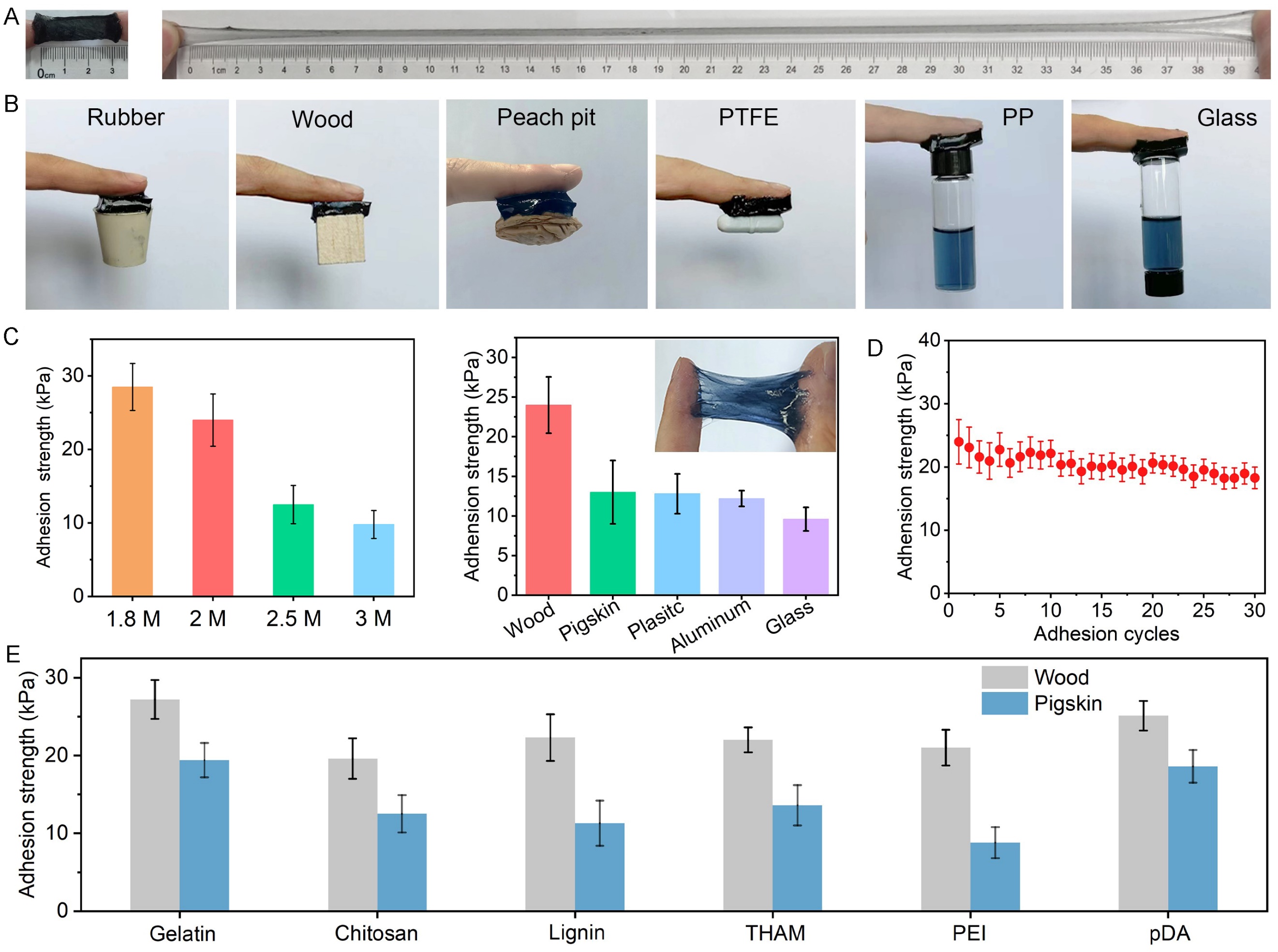


**Figure S8.** **Compliance and adhesiveness of SCMH.** A) Photos demonstrating extreme stretchability. B) Photos demonstrating high self-adhesiveness. C) Adhesive strength for hydrogels at different polymer concentrations on wood, and the 1.8 M hydrogel on different substrates. D) Adhesion stability by testing on wood as examples. E) Effect of incorporation of various additional macromolecules (gelatin, chitosan, lignin, THAM, PEI, pDA) into the hydrogels on adhesiveness.


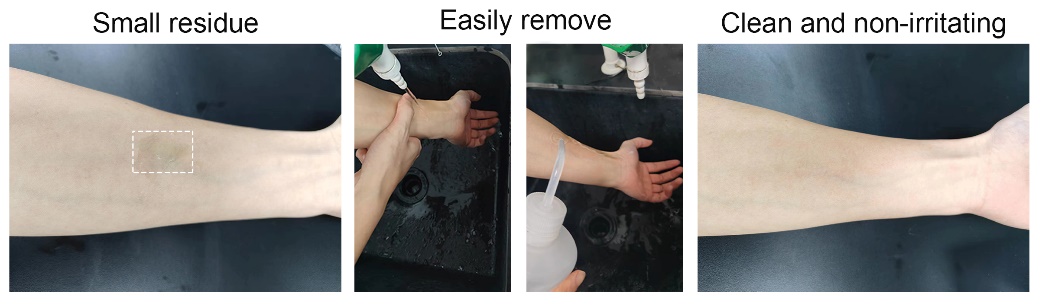


**Figure S9.** Photos depicting an ultrathin layer of residual hydrogel on the skin after peeling and its easy removal using water and ethanol.


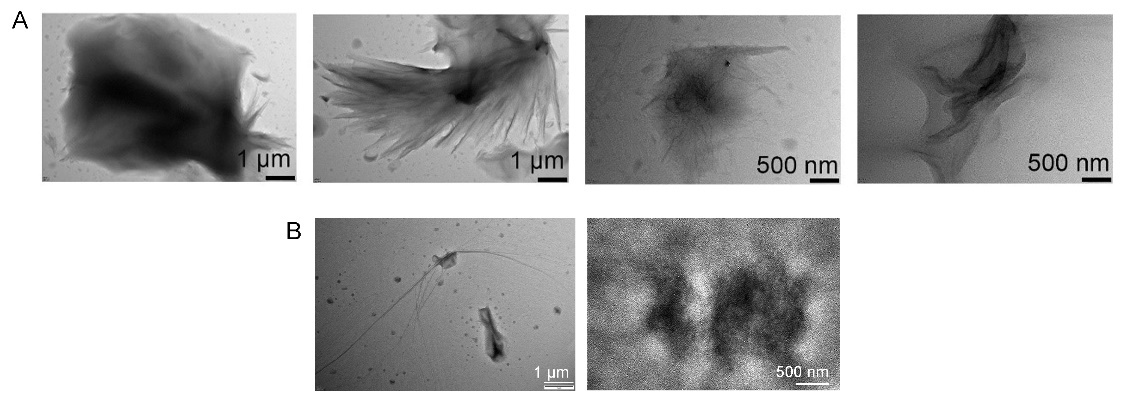


**Figure S10.** A) TEM images of CNS with various irregular membrane-like shapes prepared by dissolution and regeneration (before sulfonation). B) TEM images of SCNS (after sulfonation).


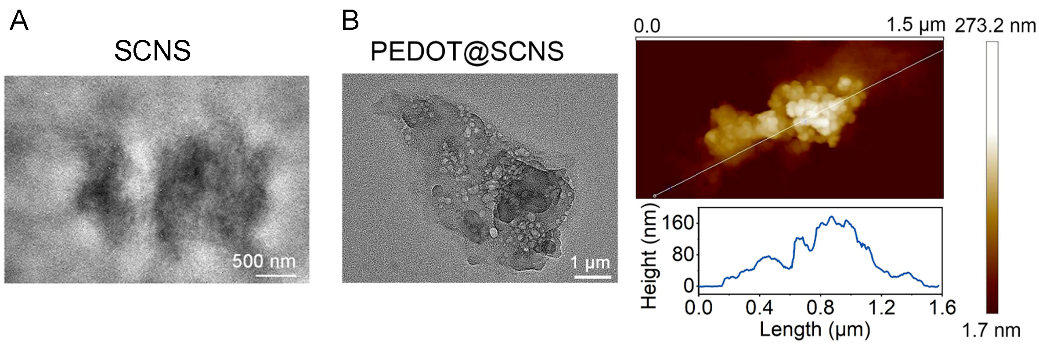


**Figure S11.** A) TEM images of SCNS (before deposition). B) TEM and AFM images of PEDOT@SCNS (after deposition).


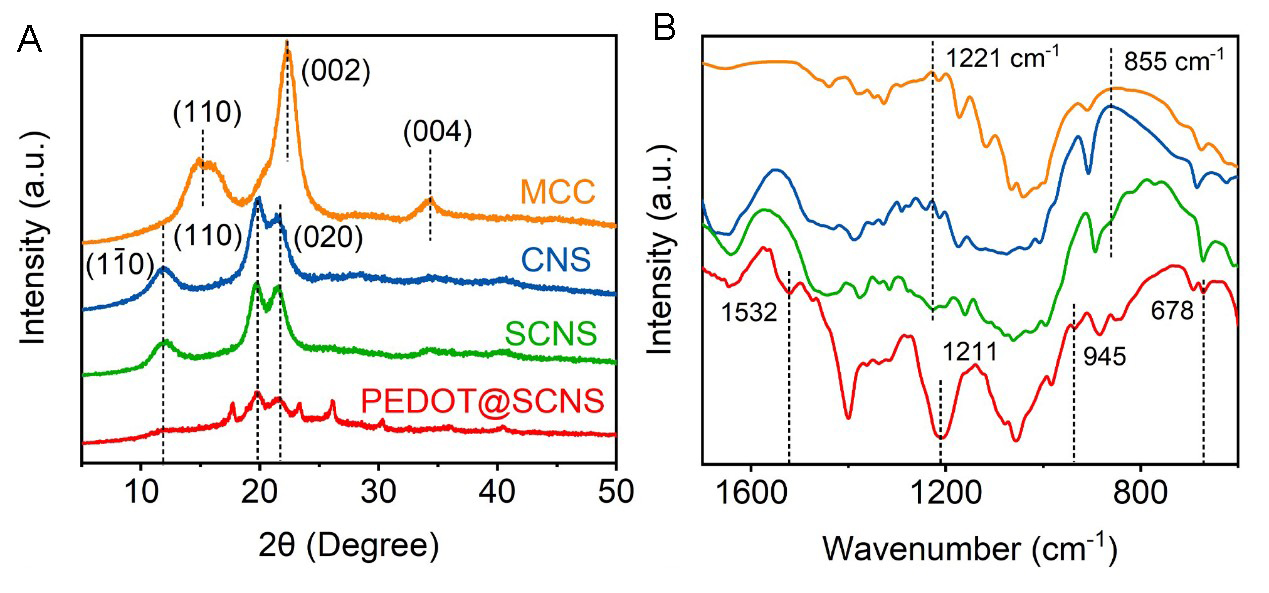


**Figure S12.** A) XRD diagrams; B) FTIR spectra of cellulose nanosheets throughout the entire preparation process.


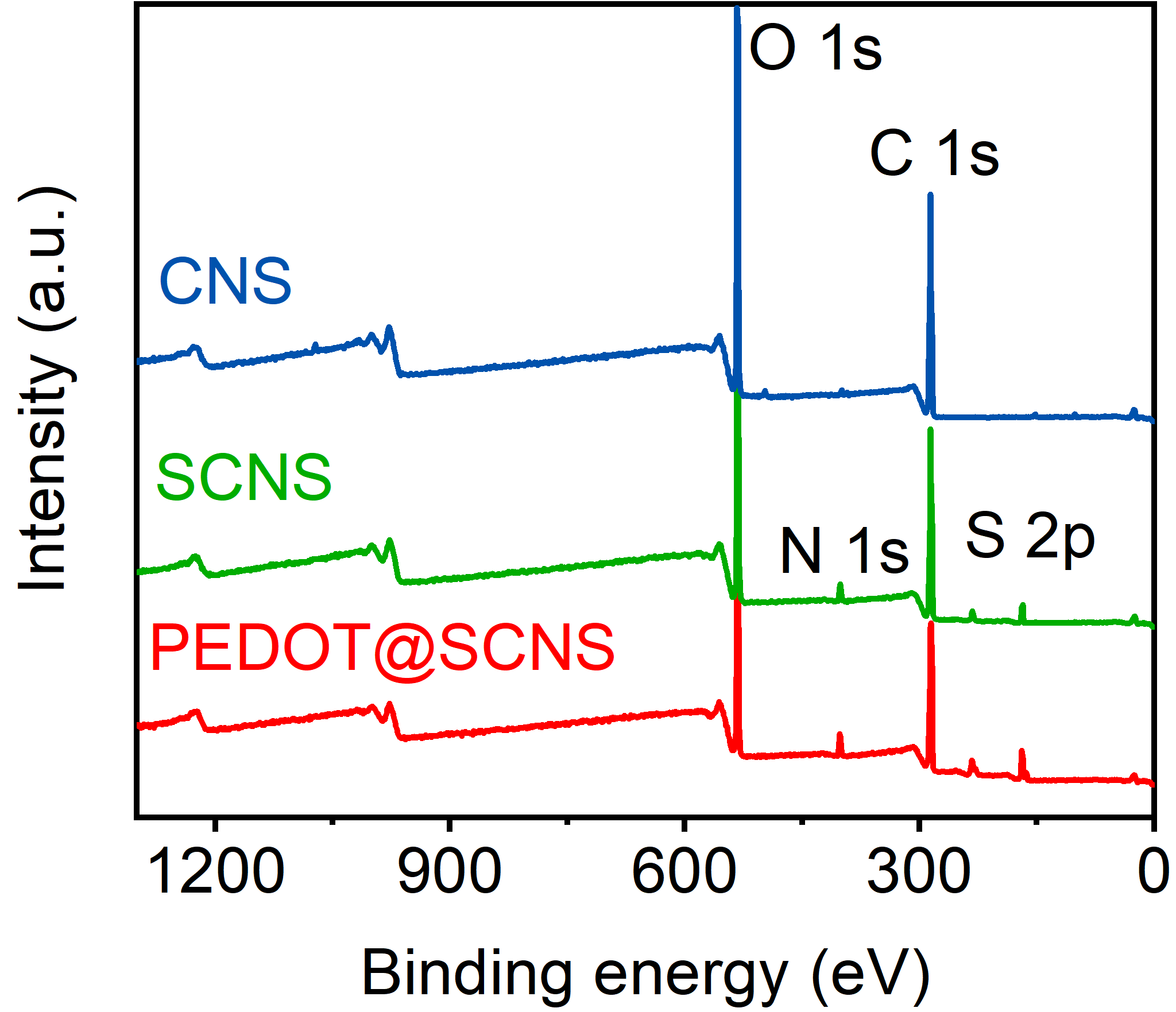


**Figure S13.** XPS spectra of the nanosheets.


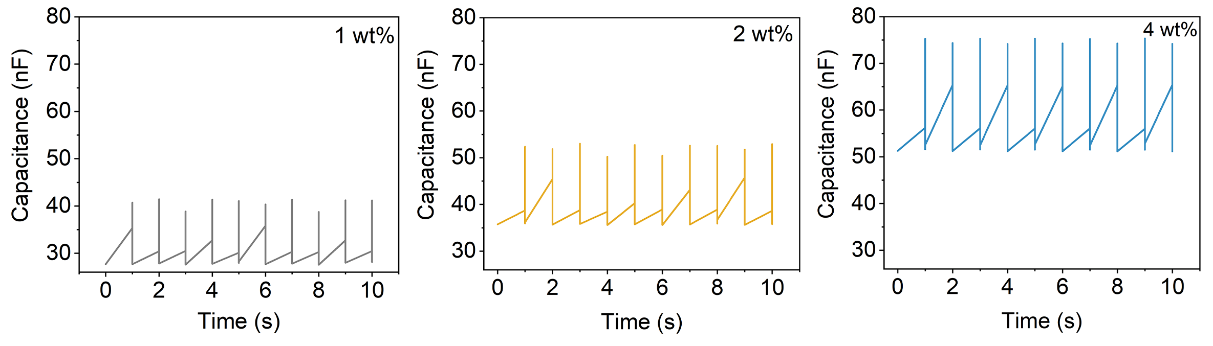


**Figure S14.** Mechanosensing performance of hydrogels with varying nanosheet contents under a 3 kPa stress and at a frequency of 1 Hz.


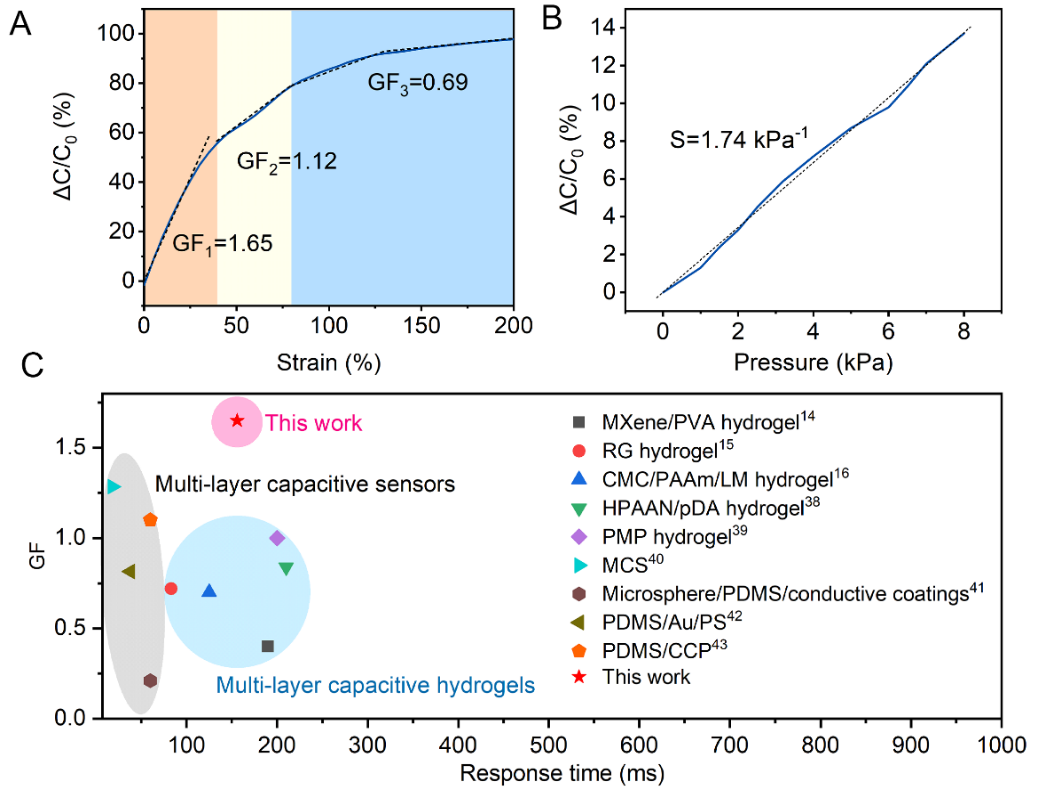


**Figure S15.** A) Relative capacitance changes *vs.* tensile strain. B) Relative capacitance changes *vs.* compressive stress. C) A comparison chart of the sensing performance with the literature.


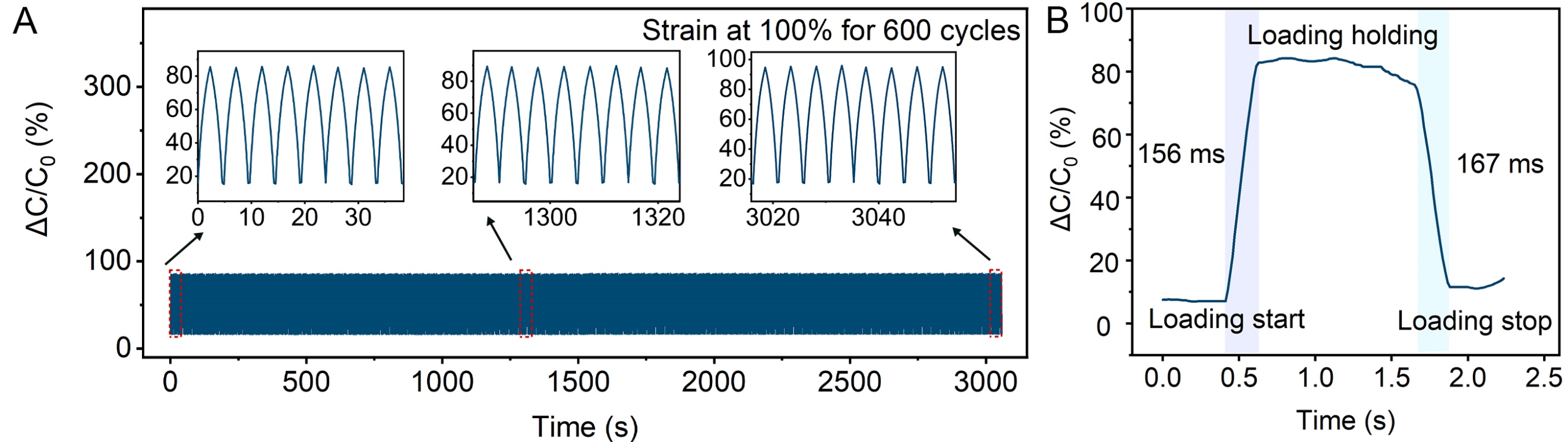


**Figure S16.** A) Long-term capacitance changes during 600 loading-unloading tensile cycles at 100% strain. B) Response and recovery time of the hydrogel sensor at 100% strain.


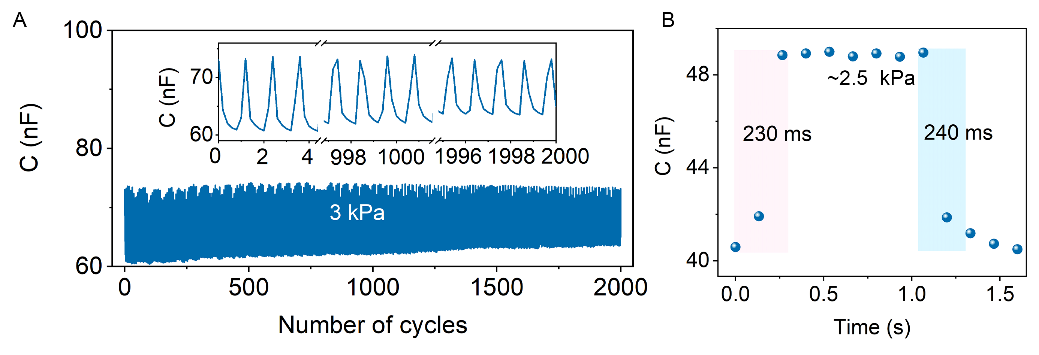


**Figure S17.** A) Cyclic compression capacitance test over 2000 cycles. B) Response time after cyclic loading.


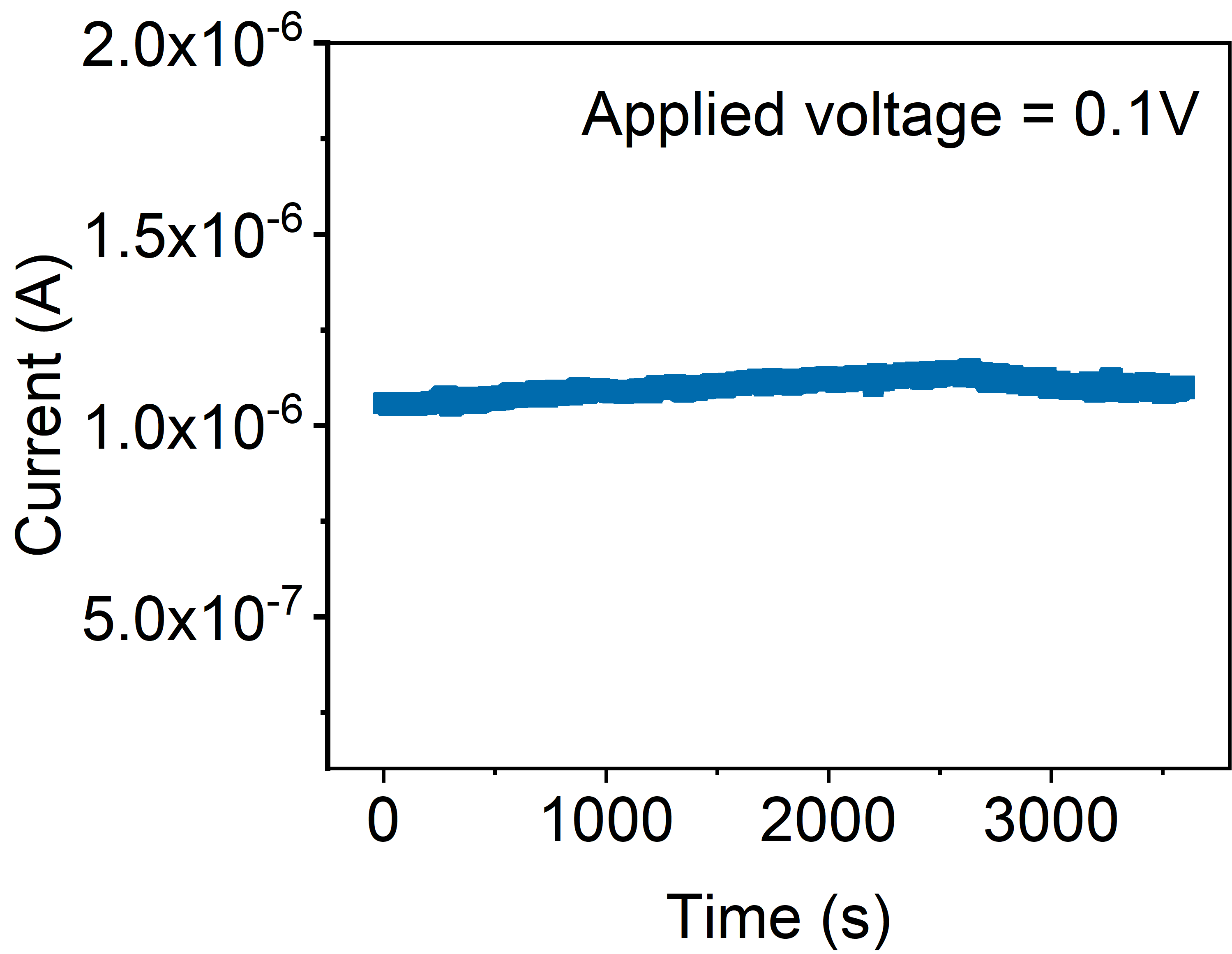


**Figure S18.** Current stability of the NSH sensor under constant voltage stimulation (0.1V) over 1h.


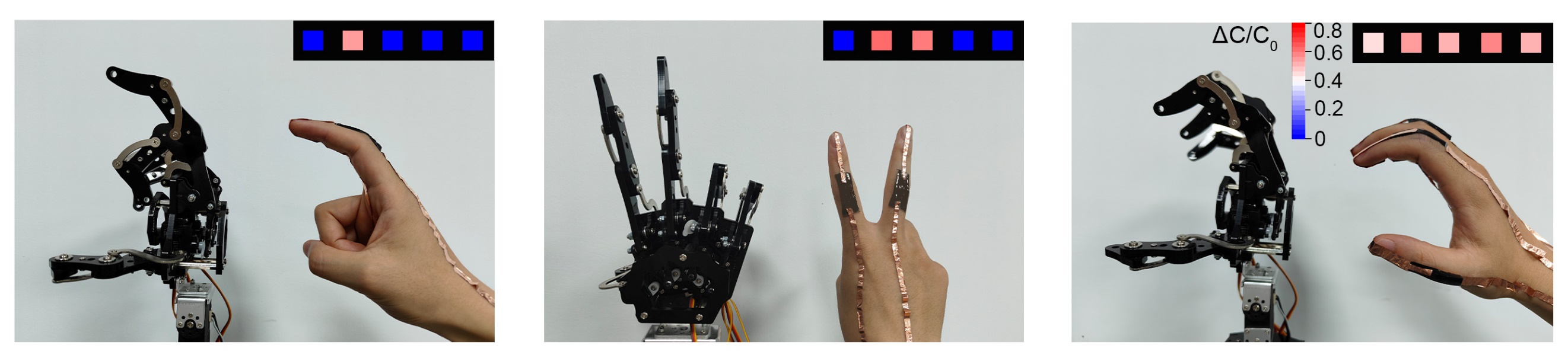


**Fig. S19.** A real-time control model for human-machine interface in operating a mechanical hand through finger bending.


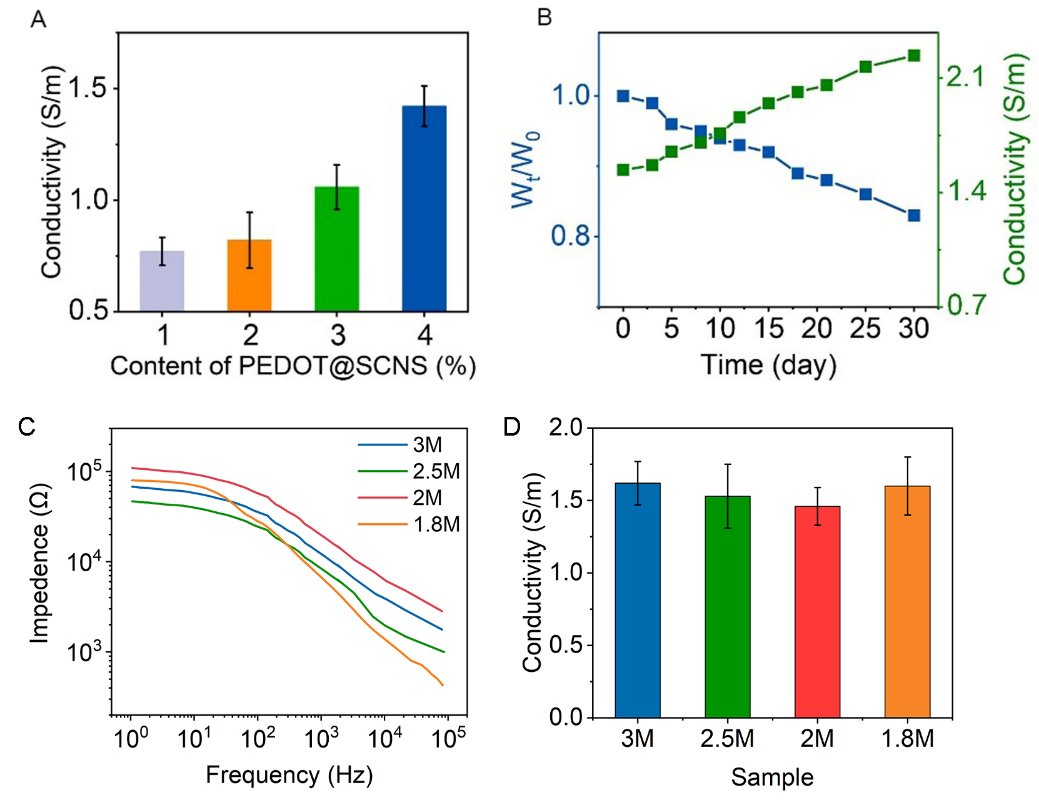


**Figure S20.** A) Effect of PEDOT@SCNS nanosheet concentration (dry mass ratio relative to AAm) on the conductivity measured by a four-point probe. B) High stability of the hydrogels demonstrated by the corresponding weight change and conductivity change (tested while encapsulated with PDMS film and then placed in air). C) Impedance spectra of hydrogels with different AAm concentrations. D) Conductivity of hydrogels with varying AAm concentrations.


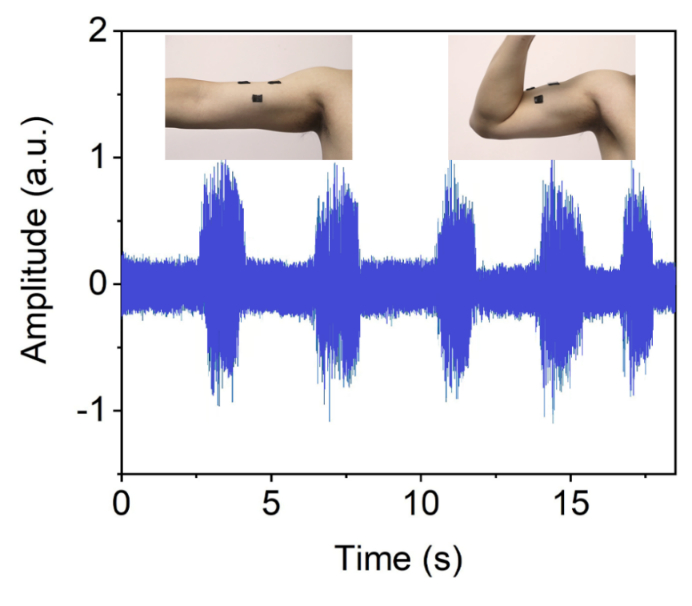


**Figure S21.** Electromyography (EMG) signals recorded from the arm.


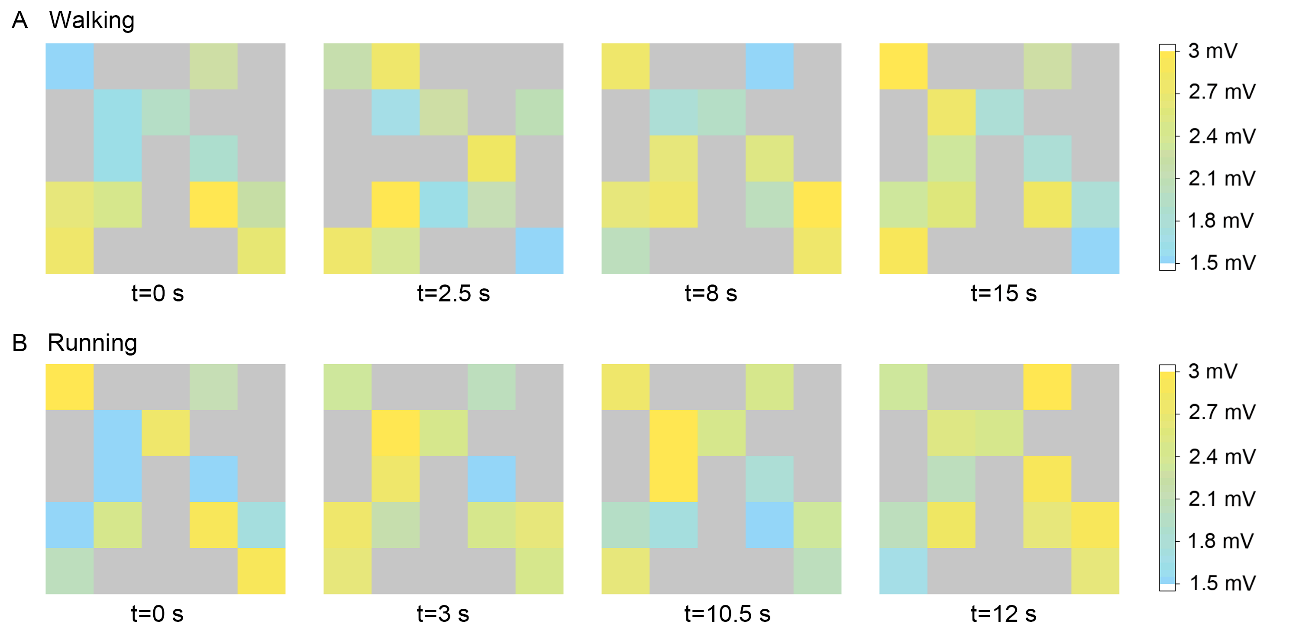


**Figure S22.** Spatiotemporal distribution array of facial EMG signals during walking and running states.


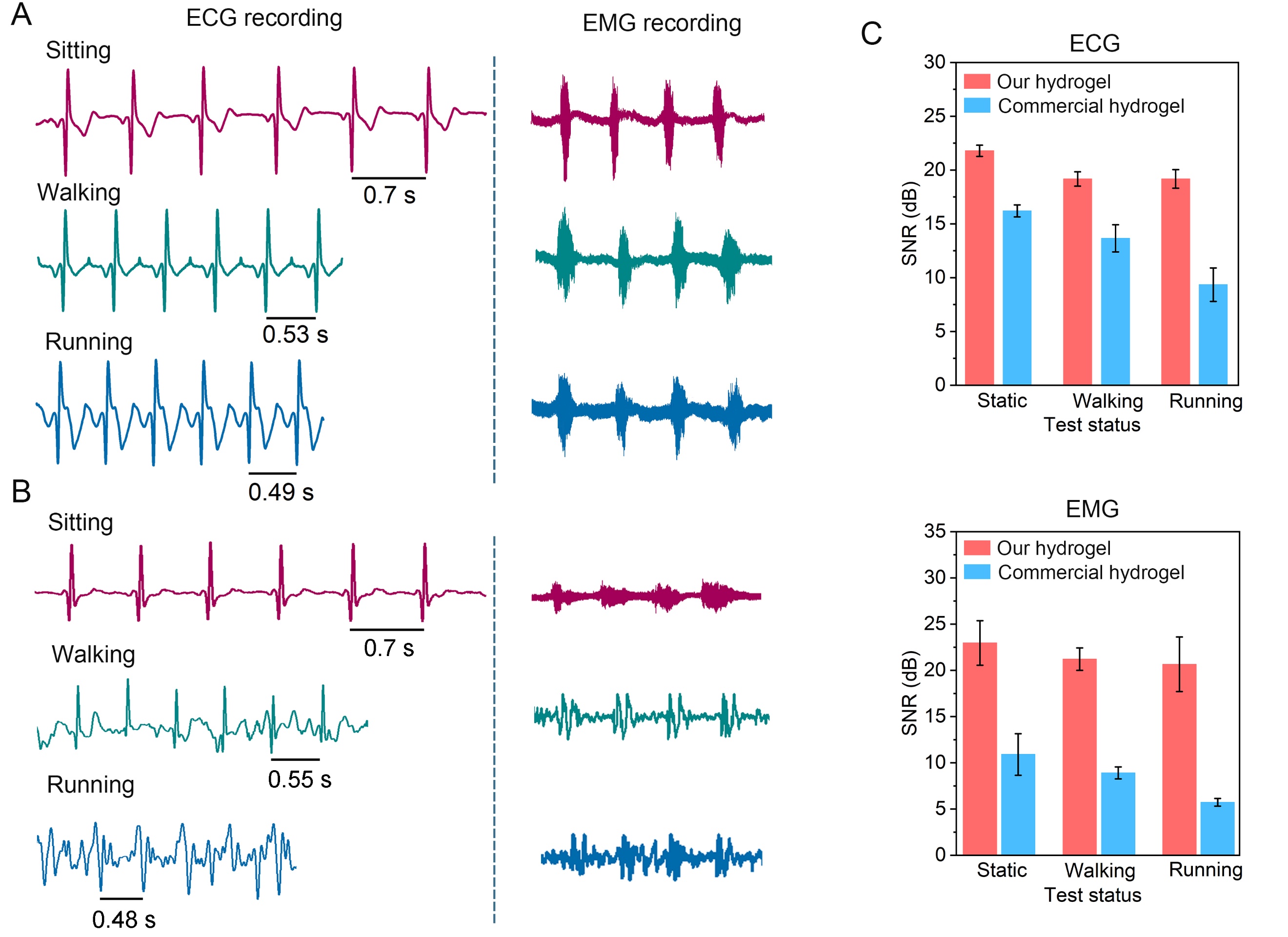


**Figure S23.** Comparison of ECG and EMG signal recording capacities during walking and running for A) our hydrogel and B) a commercial hydrogel. C) Comparison of SNR values.


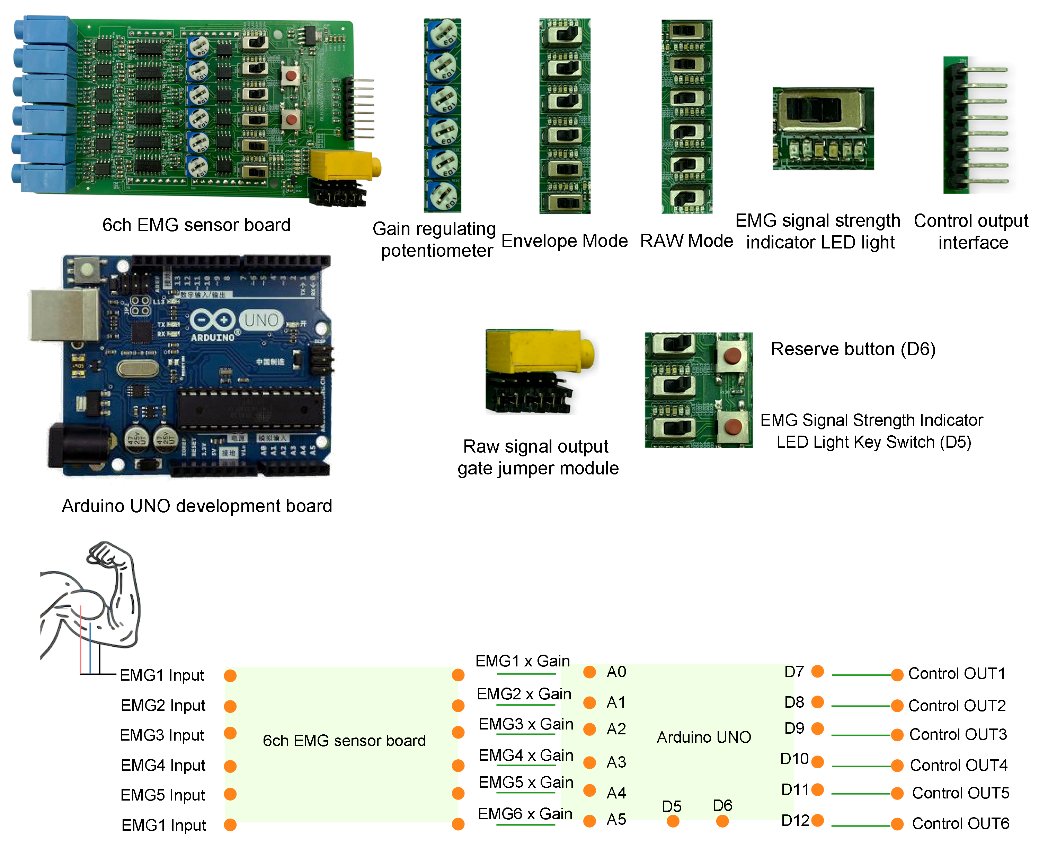


**Figure S24.** Sensor module description and function block diagram.

Table S1. Formulations of hydrogels.

| PEDOT@SCNS  (mL) | CMC  (g) | AAm  (g) | BIS  (mg) | APS  (g) | TEMED  (μL) | Gelatin  (g) | Chitosan  (g) | Lignin  (g) | THAM  (g) | PEI  (g) | pDA  (g) |
| --- | --- | --- | --- | --- | --- | --- | --- | --- | --- | --- | --- |
| 0.5 | 0.08 | 1.28 | 3.84 | 0.010 | 10 | 0 | 0 | 0 | 0 | 0 | 0 |
| 0.5 | 0.08 | 1.42 | 4.26 | 0.011 | 10 | 0 | 0 | 0 | 0 | 0 | 0 |
| 0.5 | 0.08 | 1.78 | 5.34 | 0.014 | 10 | 0 | 0 | 0 | 0 | 0 | 0 |
| 0.5 | 0.08 | 2.13 | 6.39 | 0.017 | 10 | 0 | 0 | 0 | 0 | 0 | 0 |
| 0.5 | 0.08 | 1.42 | 4.26 | 0.011 | 10 | 0.1 | 0 | 0 | 0 | 0 | 0 |
| 0.5 | 0.08 | 1.42 | 4.26 | 0.011 | 10 | 0 | 0.1 | 0 | 0 | 0 | 0 |
| 0.5 | 0.08 | 1.42 | 4.26 | 0.011 | 10 | 0 | 0 | 0.1 | 0 | 0 | 0 |
| 0.5 | 0.08 | 1.28 | 3.84 | 0.010 | 10 | 0 | 0 | 0 | 0.1 | 0 | 0 |
| 0.5 | 0.08 | 1.28 | 3.84 | 0.010 | 10 | 0 | 0 | 0 | 0 | 0.1 | 0 |
| 0.5 | 0.08 | 1.42 | 4.26 | 0.011 | 10 | 0 | 0 | 0 | 0 | 0 | 0.1 |

Table S2. Statistical details.

| Pairwise test | Tail | Comparison | T-statistic | P-value | Sig. Level | Group analysis | F-statistic | P-value |
| --- | --- | --- | --- | --- | --- | --- | --- | --- |
| Post-hoc with Bonferroni correction | Two-tailed | α wave vs θ wave | T(4) = -18.399 | 0.061 | ns | One-way repeated measures ANOVA with EEG signal as the within factor | F_(2.12)_= 1887.254 | < 0.001 |
|  |  | β wave vs θ wave | T(4) = 42.651 | < 0.001 | ** |  |  |  |
|  |  | β wave vs α wave | T(4) = 44.369 | < 0.001 | ** |  |  |  |

**Table S3.** Parameters comparison with those edge-cutting sensors.

| Entry | composition | Modulus (kPa) | Conductivity (S/m) | Adhesion strength (kPa) | GF | Durability (cycle index) | | SNR (dB) | Dynamic SNR (dB) | Biocompatibility | Application | Ref. |
| --- | --- | --- | --- | --- | --- | --- | --- | --- | --- | --- | --- | --- |
| 1 | NSH^a^ | 10^-1^ | 1.44 | 21.7 | 1.65 | 2000 | 22.9 | | 21.2 | Yes | Epidermal electronic | This work |
| 2 | PAAm^b^/TA^c^@CNFs^d^ hydrogel | 14 | 3.12 | 9 | 1.42 | – | – | | – | Yes | Strain sensor | ^[8]^ |
| 3 | PAAm/CDB^e^/PEDOT:PSS^f^ hydrogel | 3.1 | 41.6 | 65.1 | – | – | – | | – | Yes | Bioelectronic | ^[9]^ |
| 4 | SG^g^ hydrogel | 10.62 | 0.0049 | 10 | – | – | – | | – | Yes | Bioelectronic | ^[10]^ |
| 5 | PAAm/DAL^h^/rGO^i^/SA^j^ hydrogel | 0.5 | 0.056 | 14.26 | – | – | – | | – | – | Bioelectronic | ^[11]^ |
| 6 | PVA^k^/Borax/LS^l^/QHEC^m^ hydrogel | 15 | 0.25 | 7 | – | – | – | | – | – | Bioelectrode and self-powered sensor | ^[12]^ |
| 7 | PAAm/PCM^n^ hydrogels | 1 | 0.05 | 1.2 | 2.69 | 200 | – | | – | – | Strain sensor | ^[13]^ |
| 8 | MXene/PVA hydrogel | – | – | – | 0.4 | 1000 | – | | – | – | Capacitive electronic skin | ^[14]^ |
| 9 | RG^o^ hydrogel | 10 | 0.53 | – | 0.72 | 150 | – | | – | – | Capacitive sensor | ^[15]^ |
| 10 | PAAm/CMC/LM^p^ hydrogel | 10 | – | – | 0.7 | 500 | – | | – | – | Capacitive sensor | ^[16]^ |
| 11 | PAAm/PAAc^q^/N-isopropylacrylamide/pDA hydrogel | 0.4 | 27 | – | 0.84 | – | – | | – | – | Strain sensor | ^[17]^ |
| 12 | PVA/MXene/PPy^r^ hydrogel | 15 | 0.74 | 18.75 | 1 | 200 | – | | – | – | Capacitive electronic | ^[18]^ |
| 13 | PDMS^s^/Ag NWs^t^/Paraffin/Melamine sponge | – | – | – | 1.285 | 42 000 | – | | – | – | Capacitive sensor | ^[19]^ |
| 14 | PLA^u^/MWCNTs^v^ 3D-printed composite | – | – | – | 11.2 | – | – | | – | – | Capacitive sensor | ^[20]^ |
| 15 | PAAm/VSNP^w^ hydrogel | – | – | – | 16.9 | 200 | – | | – | – | Electronic skin | ^[21]^ |
| 16 | PVA/PANI^x^ hydrogel | – | – | – | 7.7 | 1000 | – | | – | – | Capacitive sensor | ^[22]^ |
| 17 | GelMA^y^/PEGDA^z^/CNT^A^/pDA hydrogel | – | 2.63 | – | – | – | 17 | | – | – | Epidermal bioelectronic | ^[23]^ |
| 18 | MXene/PAAc/ACC^B^ hydrogel | – | – | – | – | – | 19.96 | | – | Yes | Epidermal sensor | ^[24]^ |
| 19 | E-SPN^C^ bioelectrode | 5 | 52 | – | 0.02 | – | 20.9 | | 14.5 | Yes | Bioelectrode | ^[25]^ |
| 20 | PPy/leather electrode | – | – | – | – | – | 15 | | – | – | ECG electrode | ^[26]^ |
| 21 | MXene hydrogel | – | – | – | – | – | 18 | | – | – | Epidermic sensor | ^[27]^ |
| 22 | PSP^D^ hydrogel | 0.1 | 80 | 6 | – | – | 21 | | 20 | Yes | Epidermal electrode | ^[28]^ |
| 23 | PSN^E^ hydrogel | 4000 | 1850 | – | – | – | 11.4 | | – | – | Bioelectronic | ^[29]^ |
| 24 | PEDOT:PSS/PAAm/pDA hydrogel | 2.7 | 46 | 5.57 | – | – | 30 | | 21.82 | – | Physiological electrode | ^[30]^ |
| 25 | C-IPN^F^ hydrogel | 8 | 23 | – | 2.5 | – | – | | – | – | Strain electronic | ^[31]^ |
| 26 | PEDOT:PSS/STEC^G^ film | – | 41000 | – | – | – | – | | – | – | LED and FET devices | ^[32]^ |
| 27 | Supramolecular SLIC^H^ polymer | 170 | 0.012 | – | – | – | – | | – | – | Supramolecular lithium ion conductor | ^[33]^ |
| 28 | PFPE/DMA^I^ hydrogel | 30 | 47400 | – | – | – | – | | – | Yes | Nerve stimulation electrode | ^[34]^ |
| 29 | Self-healing composite | – | 4000 | – | – | – | – | | – | – | Electronic skin | ^[35]^ |
| 30 | PEDOT:PSS/PR^J^ supramolecular | 10^6^ | 270000 | – | 0.08 | 500 | – | | – | Yes | Organic bioelectronic | ^[36]^ |
| 31 | PEDOT:PSS/ECH-MEAs^K^ | 13.4 | – | – | – | – | 33.7 | | – | Yes | Physiological electrode | ^[37]^ |
| Abbreviations: ^a^super-compliant microcapacitor hydrogel (NSH); ^b^Polyacrylamide (PAAm); ^c^Tannic acid (TA); ^d^Cellulose nanofibrils (CNFs); ^e^Carboxymethyl cellulose-dopamine conjugate (CDB); ^f^Poly (3,4-ethylene dioxythiophene)-poly (styrene sulfonate) (PEDOT:PSS); ^g^Poly(sulfobetaine vinylimidazolium) (poly(SBVI))-graphene (SG); ^h^Catechol lignin (DAL); ^i^Reduced graphene oxide (rGO); ^j^Sodium alginate (SA); ^k^Polyvinyl alcohol (PVA); ^l^Lignosulfonate sodium (LS); ^m^Quaternary hydroxyethyl cellulose (QHEC); ^n^PDA coated CNC-MXene (PCM), cellulose nanocrystal (CNC); ^o^Reduced GO-enforced gelatin (RG); ^p^Liquid metal (LM); ^q^Polyacrylic acid (PAAc); ^r^Polypyrrole (PPy); ^s^Polydimethylsiloxane (PDMS); ^t^Silver nanowires (Ag NWs); ^u^Polylactic acid (PLA); ^v^Multi-walled carbon nanotubes (MWCNTs); ^w^Vinyl silica nanoparticle (VSNP); ^x^Polyaniline (PANI); ^y^Methacrylate gelatin (GelMA); ^z^Poly(ethylene-glycol) diacrylate (PEGDA); ^A^Carbon nanotube (CNT); ^B^Amorphous calcium carbonate (ACC); ^C^Supramolecular polymer networks (SPN); ^D^PEDOT:PSS/SBMA/PEGDA (PSP), [2-(methacryloyloxy)ethyl]dimethyl-(3 sulfopropyl) (SBMA); ^E^PEDOT:P(SS-co-NHMAA) (PSN), sodium 4-styrenesulfonate (SS), N-(hydroxymethyl)acrylamide (NHMAA); ^F^Conducting interpenetrating networks (C-IPN); ^G^Ionic additives–assisted stretchability and electrical conductivity (STEC); ^H^Supramolecular lithium ion conductor (SLIC); ^I^Dimethacrylate-function alized perfluoropolyether (PFPE-DMA); ^J^Polyrotaxane (PR); ^K^Electrically conductive hydrogel-micropillar electrode arrays (ECH-MEAs). | | | | | | | | | | | | |

**References**

[1] S. Nam, A. D. French, B. D. Condon, M. Concha, *Carbohydr. Polym.* **2016**, 135, 1.

[2] T. Horii, Y. Li, Y. Mori, H. Okuzaki, *Polym. J.* **2015**, 47, 695.

[3] G. Cao, S. Cai, Y. Chen, D. Zhou, H. Zhang, Y. Tian, *Polymer* **2022**, 252, 124952.

[4] F. A. J. J. o. A. Alamer, Compounds, *J. Alloys Compd.* **2017**, 702, 266.

[5] U. Heredia Rivera, S. Kadian, S. Nejati, J. White, S. Sedaghat, Z. Mutlu, R. Rahimi, *ACS Sens.* **2022**, 7, 960.

[6] Y. Wang, A. L. J. Lwal, Q. Wang, J. Zhou, A. Dufresne, N. Lin, *Chem. Commun.* **2020**, 56, 10958.

[7] a) X. Feng, X. Wang, M. Wang, S. Zhou, C. Dang, C. Zhang, Y. Chen, H. J. C. E. J. Qi, *Chem. Eng. J.* **2021**, 418, 129533.

[8] J. Lu, X. Han, L. Dai, C. Li, J. Wang, Y. Zhong, F. Yu, C. J. C. P. Si, *Carbohydr. Polym*. **2020**, 250, 117010.

[9] M. Suneetha, O. S. Moo, S. M. Choi, S. Zo, K. M. Rao, S. S. J. C. E. J. Han, *Chem. Eng. J.* **2021**, 426, 130847.

[10] I. K. Han, K. I. Song, S. M. Jung, Y. Jo, J. Kwon, T. Chung, S. Yoo, J. Jang, Y. T. Kim, D. S. J. A. M. Hwang, *Adv. Mater.* **2023**, 35, 2203431.

[11] Y. Qian, Y. Zhou, M. Lu, X. Guo, D. Yang, H. Lou, X. Qiu, C. F. J. S. M. Guo, *Small Methods* **2021**, 5, 2001311.

[12] Q. Wang, X. Pan, J. Guo, L. Huang, L. Chen, X. Ma, S. Cao, Y. J. C. E. J. Ni, *Chem. Eng. J.* **2021**, 414, 128903.

[13] B. Wan, N. Liu, Z. Zhang, X. Fang, Y. Ding, H. Xiang, Y. He, M. Liu, X. Lin, J. J. C. P. Tang, *Carbohydr. Polym*. **2023**, 314, 120929.

[14] J. Zhang, L. Wan, Y. Gao, X. Fang, T. Lu, L. Pan, F. J. A. E. M. Xuan, *Adv. Electron. Mater.* **2019**, 5, 1900285.

[15] R. Yin, C. Zhang, Y. Chen, Y. Wang, Q. Feng, Y. Liu, M. Yu, Y. Yuan, C.-Y. Xu, F. J. C. E. J. Liu, *Chem. Eng. J.* **2023**, 475, 145794.

[16] R. Tang, Q. Meng, Z. Wang, C. Lu, M. Zhang, C. Li, Y. Li, X. Shen, Q. J. A. a. m. Sun, interfaces, *ACS Appl. Mater. Interfaces* **2021**, 13, 57725.

[17] Z. Gao, L. Kong, R. Jin, X. Liu, W. Hu, G. J. J. o. M. C. C. Gao, *J. Mater. Chem. C* **2020**, 8, 11119.

[18] Z. Qin, G. Zhao, Y. Zhang, Z. Gu, Y. Tang, J. T. Aladejana, J. Ren, Y. Jiang, Z. Guo, X. J. S. Peng, *Small* **2023**, 19, 2303038.

[19] M. O. Cicek, D. Doganay, M. B. Durukan, M. C. Gorur, H. E. J. A. m. t. Unalan, *Adv. Mater. Technol.* **2021**, 6, 2001168.

[20] S. Matsalis, G. Paterakis, N. Koutroumanis, G. Anagnostopoulos, C. J. S. I. Galiotis, *Sensors Int*. **2024**, 5, 100272.

[21] Y. Cai, J. Shen, C.-W. Yang, Y. Wan, H.-L. Tang, A. A. Aljarb, C. Chen, J.-H. Fu, X. Wei, K.-W. J. S. a. Huang, *Sci. Adv.* **2020**, 6, eabb5367.

[22] H. Zhou, M. Wang, X. Jin, H. Liu, J. Lai, H. Du, W. Chen, A. J. A. A. M. Ma, Interfaces, *ACS Appl. Mater. Interfaces* **2021**, 13, 1441.

[23] H. Tang, Y. Li, B. Chen, X. Chen, Y. Han, M. Guo, H.-q. Xia, R. Song, X. Zhang, J. J. A. n. Zhou, *ACS Nano* **2022**, 16, 17931.

[24] X. Li, L. He, Y. Li, M. Chao, M. Li, P. Wan, L. J. A. n. Zhang, *ACS Nano* **2021**, 15, 7765.

[25] S. J. O'Neill, Z. Huang, M. H. Ahmed, A. J. Boys, S. Velasco‐Bosom, J. Li, R. M. Owens, J. A. McCune, G. G. Malliaras, O. A. J. A. M. Scherman, *Adv. Mater*. **2023**, 35, 2207634.

[26] K. Zhang, N. Kang, B. Zhang, R. Xie, J. Zhu, B. Zou, Y. Liu, Y. Chen, W. Shi, W. J. A. E. M. Zhang, *Adv. Electron. Mater.* **2020**, 6, 2000259.

[27] M. Li, Y. Zhang, L. Lian, K. Liu, M. Lu, Y. Chen, L. Zhang, X. Zhang, P. J. A. F. M. Wan, *Adv. Funct. Mater*. **2022**, 32, 2208141.

[28] X. Huang, C. Chen, X. Ma, T. Zhu, W. Ma, Q. Jin, R. Du, Y. Cai, M. Zhang, D. J. A. F. M. Kong, *Adv. Funct. Mater*. **2023**, 33, 2302846.

[29] J. Yu, F. Tian, W. Wang, R. Wan, J. Cao, C. Chen, D. Zhao, J. Liu, J. Zhong, F. J. C. o. M. Wang, *Chem. Mater.* **2023**, 35, 5936.

[30] R. Wan, J. Yu, Z. Quan, H. Ma, J. Li, F. Tian, W. Wang, Y. Sun, J. Liu, D. J. C. E. J. Gao, *Chem. Eng. J.* **2024**, 151454.

[31] V. R. Feig, H. Tran, M. Lee, Z. J. N. c. Bao, *Nature Comm.* **2018**, 9, 2740.

[32] Y. Wang, C. Zhu, R. Pfattner, H. Yan, L. Jin, S. Chen, F. Molina-Lopez, F. Lissel, J. Liu, N. I. J. S. a. Rabiah, *Sci. Adv.* **2017**, 3, e1602076.

[33] D. G. Mackanic, X. Yan, Q. Zhang, N. Matsuhisa, Z. Yu, Y. Jiang, T. Manika, J. Lopez, H. Yan, K. J. N. c. Liu, *Nature Comm.* **2019**, 10, 5384.

[34] Y. Liu, J. Liu, S. Chen, T. Lei, Y. Kim, S. Niu, H. Wang, X. Wang, A. M. Foudeh, J. B.-H. J. N. b. e. Tok, *Nat. Biomed. Eng.* **2019**, 3, 58.

[35] B. C. Tee, C. Wang, R. Allen, Z. J. N. n. Bao, *Nature Nanotech.* **2012**, 7, 825.

[36] Y. Jiang, Z. Zhang, Y.-X. Wang, D. Li, C.-T. Coen, E. Hwaun, G. Chen, H.-C. Wu, D. Zhong, S. J. S. Niu, *Science* **2022**, 375, 1411.

[37] Y. Liu, A. F. McGuire, H.-Y. Lou, T. L. Li, J. B.-H. Tok, B. Cui, Z. J. P. o. t. N. A. o. S. Bao, *Proc. Natl. Acad. Sci.* **2018**, 115, 11718.

[38] Z. Gao, L. Kong, R. Jin, X. Liu, W. Hu, G. J. J. o. M. C. C. Gao, *J. Mater. Chem. C* **2020,** 8 (32), 11119.

[39] Z. Qin, G. Zhao, Y. Zhang, Z. Gu, Y. Tang, J. T. Aladejana, J. Ren, Y. Jiang, Z. Guo, X. J. S. Peng, *Small* **2023,** 19 (45), 2303038.

[40] M. O. Cicek, D. Doganay, M. B. Durukan, M. C. Gorur, H. E. J. A. m. t. Unalan, *Adv. Mater. Technol.* **2021,** 6 (6), 2001168.

[41] T. Shao, J. Wu, Y. Zhang, Y. Cheng, Z. Zuo, H. Lv, M. Ying, C. Wong, Z. J. A. M. T. Li, *Adv. Mater. Technol.* **2020,** 5 (5), 2000032.

[42] T. Li, H. Luo, L. Qin, X. Wang, Z. Xiong, H. Ding, Y. Gu, Z. Liu, T. J. S. Zhang, *Small* **2016,** 12 (36), 5042.

[43] P. Wei, X. Guo, X. Qiu, D. J. N. Yu, *Nanotechnology* **2019,** 30 (45), 455501.
